# Multi‐omics analyses reveal epigenomics basis for cotton somatic embryogenesis through successive regeneration acclimation process

**DOI:** 10.1111/pbi.12988

**Published:** 2018-09-30

**Authors:** Jianying Li, Maojun Wang, Yajun Li, Qinghua Zhang, Keith Lindsey, Henry Daniell, Shuangxia Jin, Xianlong Zhang

**Affiliations:** ^1^ National Key Laboratory of Crop Genetic Improvement Huazhong Agricultural University Wuhan Hubei China; ^2^ Department of Biosciences Durham University Durham UK; ^3^ Department of Biochemistry School of Dental Medicine University of Pennsylvania Philadelphia PA USA

**Keywords:** cotton, somatic embryogenesis, SRA, plant regenerative ability, DNA methylation, RdDM, H3K9me2

## Abstract

Plant regeneration via somatic embryogenesis is time‐consuming and highly genotype‐dependent. The plant somatic embryogenesis process provokes many epigenetics changes including DNA methylation and histone modification. Recently, an elite cotton Jin668, with an extremely high regeneration ability, was developed from its maternal inbred Y668 cultivar using a Successive Regeneration Acclimation (SRA) strategy. To reveal the underlying mechanism of SRA, we carried out a genome‐wide single‐base resolution methylation analysis for nonembryogenic calluses (NECs), ECs, somatic embryos (SEs) during the somatic embryogenesis procedure and the leaves of regenerated offspring plants. Jin668 (R4) regenerated plants were CHH hypomethylated compared with the R0 regenerated plants of SRA process. The increase in CHH methylation from NEC to EC was demonstrated to be associated with the RNA‐dependent DNA methylation (RdDM) and the H3K9me2‐dependent pathway. Intriguingly, the hypomethylated CHH differentially methylated regions (DMRs) of promoter activated some hormone‐related and *WUSCHEL*‐related homeobox genes during the somatic embryogenesis process. Inhibiting DNA methylation using zebularine treatment in NEC increased the number of embryos. Our multi‐omics data provide new insights into the dynamics of DNA methylation during the plant tissue culture and regenerated offspring plants. This study also reveals that induced hypomethylation (SRA) may facilitate the higher plant regeneration ability and optimize maternal genetic cultivar.

## Introduction

Somatic embryogenesis is a cell differentiation process and is involved in dedifferentiation and redifferentiation through reconstruction of somatic cells to generate embryogenic cells (EC) (Yang and Zhang, 2010). Somatic cells undergo dedifferentiation to generate nonembryogenic cells (NEC) from diverse explants, including hypocotyls, young leaves and immature embryos. NEC cells can further dedifferentiate to generate EC cells and play a role in somatic embryo development, which includes three stages: globular embryos, torpedo embryos and cotyledon embryos. This process can be used to regenerate new plants (Leelavathi *et al*., 2004). The mechanisms of gene regulation during the tissue culture and somatic embryogenesis process have been investigated in several crops, such as soybean, potato and cotton (Sharma *et al*., 2008; Thibaud‐Nissen *et al*., 2003; Yang *et al*., 2012). This somatic embryogenesis process will encounter epigenetic variation, such as noncoding RNAs, DNA methylation and histone modification, with effects on gene expression (De‐la‐Pena *et al*., 2015).

DNA methylation of cytosine base is one of the most extensively studied heritable epigenetic modifications in eukaryotes. DNA methylation plays essential roles in many biological processes, such as genomic imprinting, transposon element (TEs) silencing, heterochromatin maintenance and X‐chromosome inactivation (Chen, 2013; Hatorangan *et al*., 2016; Slotkin *et al*., 2009). In plants, DNA methylation can be categorized into three contexts: symmetric CG and CHG (where H = A, T or C), and asymmetric CHH (Henderson and Jacobsen, 2007). In *Arabidopsis*, CG methylation is maintained by a conserved METHYLTRANSFERASE1 (*MET1*) (homologous to animal *DNMT1*) protein. CHG and CHH methylation are primarily maintained by the plant‐specific CHROMOMETHYLASE3 (*CMT3*) and *CMT2*, respectively (Law and Jacobsen, 2010). DNA methylation in all contexts is *de novo* established by a 24‐nt small interfering RNA (siRNA) directed DNA methylation pathway (RdDM) involving the DOMAINS REARRANGED METHYLTRANSFERASE1 (*DRM1*) and *DRM2* in plants (Mosher and Melnyk, 2010). The canonical RdDM pathway requires two plant‐specific RNA polymerases, Pol IV and Pol V. The biogenesis of 24‐nt siRNAs requires Pol IV, RNA‐dependent RNA polymerase 2 (*RDR2*) and Dicer‐like 3 (*DCL3*). Non‐CG (including CHG and CHH) methylation could also be mediated independently of the RdDM pathway through DECREASED DNA METHYLATION 1 (*DDM1*) and *CMT2/3* involving of the dimethylation of histone H3 at lysine 9 (H3K9me2) in heterochromatin regions (Du *et al*., 2012; Stroud *et al*., 2014). *DDM1* is a chromatin‐remodelling factor in *Arabidopsis* (Zemach *et al*., 2013). Also, RdDM and *DDM1*‐involved pathways synergistically mediate non‐CG methylation and collaborate to prevent gene transcription (Zemach *et al*., 2013). In addition, it is known that active demethylation is mediated by the REPRESSOR OF SILENCING 1 (*ROS1*) and DNA glycosylase DEMETER (*DME*) enzymes (Gehring *et al*., 2006; Gong *et al*., 2002).

In *Arabidopsis*, the use of the McrBC‐digested DNA tiling array has shown that DNA methylation patterns distinctly changed in cell suspension culture when compared with wild‐type plants (Tanurdzic *et al*., 2008). Furthermore, the regenerated rice lines from tissue culture exhibited hypomethylation compared with WT plants (Stroud *et al*., 2013). The decrease in DNA methylation in promoter regions is heritable and is associated with the up‐regulated expression of nearby genes. In contrast, some DNA methylation sites were altered among callus cell lines, regenerated plants and the regenerated‐derived progeny of maize (Stelpflug *et al*., 2014). Interestingly, the DNA methylation of specific TE families (*LORE1a*) is altered and demethylation can result in transcription reactivation during plant tissue culture processes in model *legume Lotus japonicus* (Fukai *et al*., 2010). In oil palm, the loss methylation and small RNA of *LINE* retrotransposon contribute to the origin of mantled during tissue culture (Ong‐Abdullah *et al*., 2015). However, very limited studies have focused on the global DNA methylation changes in genome‐wide single‐base resolution during tissue culture process and regenerated offspring plants.

Cotton (*Gossypium spp*.) is a widely cultivated crop and has long been recognized as an important resource for renewable textile fibres. Despite this, further trait improvement requires efficient genetic transformation procedures. Tissue culture and genetic modifications are extensively applied to cotton breeding, such as the modifications that have produced pest resistance (Wilkins *et al*., 2000). The standard procedure of cotton tissue culture has also been successfully used for functional genome analysis (Jin *et al*., 2006a). However, the regeneration ability of cotton is highly genotype‐dependent. Only a few genotypes can generate regenerated plants through somatic embryogenesis including Cocker lines (Cocker 201/310/312), Simian 3 and Jihe 321 (Wu *et al*., 2004). Previously, an elite genotype YZ1 was identified and widely used for cotton tissue culture and genetic transformation in our laboratory, which showed higher regeneration ability (Jin *et al*., 2006b). Unfortunately, all of these genotypes were developed more than 20–30 years ago, and therefore, their agronomic traits are quite poor and outdated. An elite cotton genotype with a higher regenerative ability and ideal agronomic traits is highly desirable for cotton biotechnology and molecular breeding. Given the increasing adoption of transgenic crops worldwide, it is desirable to find more highly regenerate genotypes. Recently, a newly elite cotton genotype, Jin668, was developed in our laboratory using a Successive Regeneration Acclimation (SRA) strategy and now widely used for the cotton genetic transformation in our group (Luo *et al*., 2017; Wang *et al*., 2017). However, the molecular mechanism underlying this phenomenon is not clear. It is well known that the process of plant tissue culture affects the expression of many genes, which are in turn regulated by diverse epigenetic mechanisms, especially DNA methylation and histone modification (Ikeuchi *et al*., 2016; Miguel and Marum, 2011). Until now, the effects of DNA methylation dynamics on gene expression changes in the tissue culture process and their regenerated offspring plants have not been investigated in cotton.

Here, we explored DNA methylation dynamics during the SRA process for the identification of the elite Jin668 and the entire cotton tissue culture process using Bisulphite‐treated sequencing (BS‐Seq). We found that there were great differences in the DNA methylation patterns in different stages of the tissue culture process and in successive generations of regenerated plants. Furthermore, analysis of differentially methylated regions (DMRs) showed that some DMRs could regulate the expression of nearby functional genes. This study provides a feasible strategy to improve the regenerative ability of plants and produce novel insights into the effects of dynamic DNA methylation on cell differentiation and plant regeneration.

## Results

### Developing the elite “Jin668” for cotton genetic transformation from maternal cultivar “Y668” by a successive regeneration acclimation (SRA) strategy

**Figure 1:**
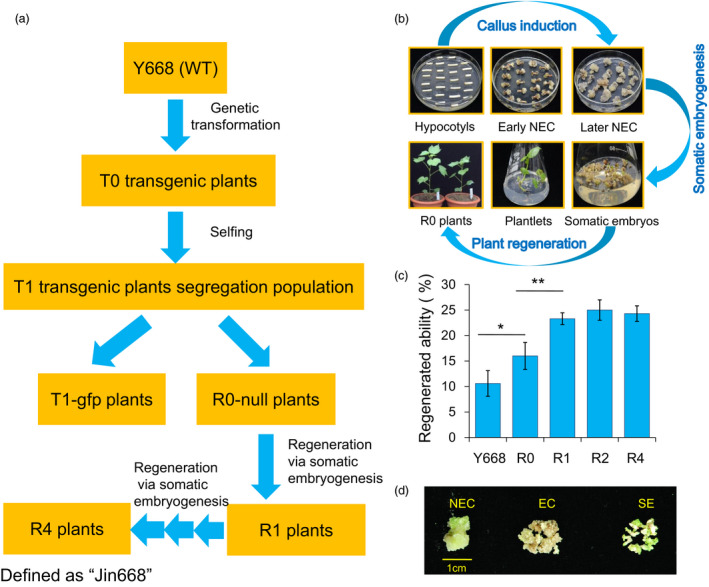
Developing the elite “Jin668” from the maternal cultivar Y668 using the Successive Regeneration Acclimation (SRA) strategy. (a) Schematic diagram of SRA strategy with wild‐type (WT) cotton plants as maternal cultivar. (b) The standard process of *Agrobacterium‐*mediated genetic transformation and plant regeneration via somatic embryogenesis in cotton. NEC: nonembryogenic callus; EC: embryogenic callus; SE: somatic embryo. (c) The comparison of regeneration ability from different generations in SRA process (the number of explants producing embryogenic callus per 100 explants within 4‐month tissue culture process (Student's *t*‐test, *P <* *0.05, **P <* *0.01). (d) Different stages of cotton somatic embryogenesis. These calluses represent 75‐day NEC, EC and SE used for Bisulphite‐treated sequencing.

The hypocotyls of maternal inbred Y668 cotton plantlets were used as explants for *Agrobacterium‐*mediated genetic transformation with *GFP* as a reporter gene. The seeds from regenerated plants (R0) were screened by PCR and *GFP* fluorescence detection. The negative plants (null, R0 progeny) were selected for further use. The hypocotyls from seeds of R0 plants were used for tissue culture and somatic embryogenesis to generate the R1 generation. Then, this regeneration process was repeated thrice to generate the R2, R3 and R4 generations (Figure 1a). The R4 generation was then defined as Jin668, and this whole process was defined as the Successive Regeneration Acclimation (SRA) strategy. The whole cotton tissue culture and somatic embryogenesis process were streamlined in Figure 1b. We compared the regenerative efficiency of Y668 (WT), R0, R1, R2 and R4 (Jin668), and the results showed that Jin668 gained 2.2 times higher regenerative ability after SRA compared with the maternal inbred cultivar Y668 and up to six times higher regenerative ability (20%–25% vs 3%–5%) than the most widely adopted cotton varieties Coker 310/312 (Figure 1c).

### Genome‐wide DNA methylation profiles between somatic embryogenesis and regenerated plants during the SRA process

To investigate the dynamic patterns of DNA methylation during the somatic embryogenesis process and in the regenerated plants, Bisulphite‐treated sequencing (BS‐Seq) was carried out to construct high‐resolution maps. DNA methylation profiles of regenerated plants during the SRA process were investigated. Cotton leaves from regenerated plants of three generations (R0, R2 and R4) during the SRA process and maternal plants (Y668) were collected for BS‐Seq (Figure 1a). Moreover, calluses at three developmental stages, namely NEC (75 days (d)), EC and SE derived from Jin668, were also collected for DNA methylation analysis (Figure 1d).

**Figure 2:**
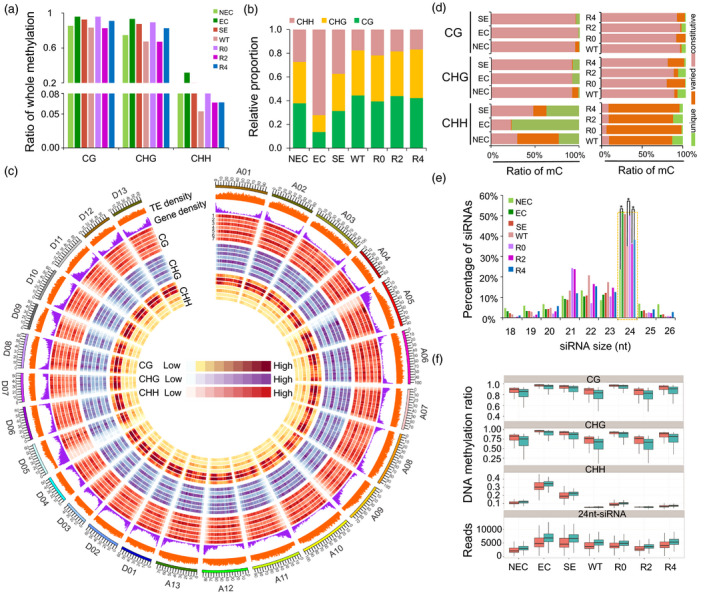
Genomic landscape of DNA methylation in cotton somatic embryogenesis process and regenerated plants. (a) Percentages of methylated cytosines (mCs) in the somatic embryogenesis and regenerated plants. (b) The relative content of mCs in CG, CHG and CHH contexts from seven samples. (c) Circos plot showing gene density, TE density, ratios of CG, CHG and CHH methylation in callus and leaves of regenerated plants using 1‐Mb sliding 200‐kb windows among *A*_*t*_‐subgenome chromosomes (right) and *D*_*t*_‐subgenome chromosomes (left). The outer track represents the 26 chromosomes of the *G. hirsutum* genome. Tracks 1–7 represent NEC, EC, SE, WT, R0, R2 and R4, respectively. (d) Comparison of differentially methylated cytosines (DMCs) in CG, CHG and CHH contexts in NEC, EC, SE and regenerated plants (R0, R2 and R4). Methylated cytosines observed in callus of three stages of somatic embryogenesis and all regenerated plant leaf are referred to as constitutive cytosines. Methylated cytosines observed in two or three stages/regenerated plants are referred to as varied cytosines. Methylated cytosines observed in only one stage/regenerated plant leaf are referred to as unique cytosines. (e) Percentages of unique siRNAs in the callus and leaf tissues. The 24‐nt unique siRNAs are shown in the green dotted box (Student's *t*‐test *P <* *0.05). (f) Comparison of DNA methylation and 24‐nt siRNAs in the *A*_*t*_‐ (red) and *D*_*t*_‐subgenome (green). *A*_*t*_ and *D*_*t*_ indicate the *A*_*t*_‐ and *D*_*t*_‐subgenome in the allotetraploid cotton, respectively.

Clean data were mapped against the *G. hirsutum* genome (Zhang *et al*., 2015) to identify methylated cytosines (Table S1). For each sample, approximately 60%–72% of the clean reads were uniquely mapped and ~240–320 million cytosines supported by at least three reads were covered. A total of 54–210 million methylated cytosines (mCs) were identified from these seven samples (Table S2). In the mCs, the ratios of methylated cytosines in the CG and CHG contexts were higher than in the CHH context (Figure 2a). In NEC, EC and SE, ~27%, ~72% and ~38% of methylated cytosines were in the CHH context, respectively, while ~38%, ~14%, ~31% and ~35%, ~14%, ~31% were in the CG and CHG contexts (Figure 2b). In regenerated plant leaves during the SRA process, the ratios of the CG and CHG contexts were much higher than those in callus derived from Jin668.

The DNA methylation landscape at the chromosomal level revealed that the majority of mCs were predominantly enriched in the some chromosomes centromeric regions (Figure 2c). Overall, the CG and CHG methylation levels were similar during different somatic embryogenesis stages, and the CHH methylation level significantly increased during the transition from the NEC to the EC stage. Based on these DNA methylation maps, we analysed the differential methylation of cytosines (DMCs) during somatic embryogenesis and regenerated plant leaves during the SRA process (Figure 2d). In callus developmental stages, more than 92% of cytosines were constitutively methylated in the CG and CHG contexts during the NEC, EC and SE stages, while only 22%–47% of cytosines were methylated in the CHH context at these three stages (Figure 2d). Furthermore, more unique mCHHs have been identified in the EC and SE stages (77% and 37%, respectively), whereas less unique mCHHs were identified in regenerated plants (Figure 2d). These results indicated that the regenerated plants have decreased DNA methylation compared with R0 during the SRA process.

DNA methylation is established *de novo* by the DOMAINS REARRANGED METHYLTRANSFERASE (*DRM*) protein through an RNA‐directed DNA methylation (RdDM) pathway (Matzke and Mosher, 2014), which requires the involvement of 24‐nt siRNAs. Therefore, small RNA sequencing was performed using samples from tissue culture (NEC, EC, SE), regenerated plant leaves (R0, R2, R4) and WT (Table S3). These data showed that the enrichment pattern of 24‐nt siRNAs was similar to the trend of CHH methylation during the tissue culture process (Figure 2e, Student's *t*‐test, *P <* *0.05). Meanwhile, DNA methylation levels in the symmetric CG and CHG contexts of the *A*_*t*_‐subgenome were higher than those in the *D*_*t*_‐subgenome, whereas methylation levels in the CHH context and enrichment of 24‐nt siRNAs of *D*_*t*_‐subgenome were remarkably higher than those in the *A*_*t*_‐subgenome (Figure 2f).

### DNA methylation profiles between maternal Y668 and regenerated R0, R2 and R4 plants during the SRA process

**Figure 3:**
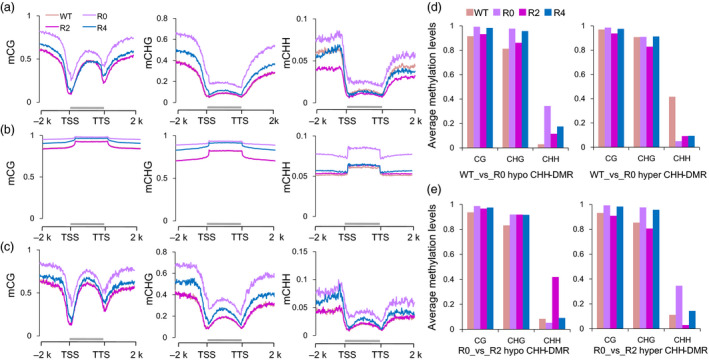
DNA methylation changes between Y668 (WT) and regenerated plants. (a‐c) Average cytosine methylation levels of protein‐coding genes (PCGs) (a), transposon elements (TEs) (b) and TE‐related genes (TEGs) (c) in CG, CHG and CHH contexts. The PCGs, TEs, TEGs are scaled of their transcriptional start sites (TSSs) and transcriptional termination sites (TTSs). Methylation levels of PCGs, TEs, TEGs with their flanking 2‐kb upstream and downstream regions were calculated in 100 intervals. (d) Average CG, CHG and CHH of hypo‐ and hyper‐CHH‐DMRs in the comparison of WT_vs_R0. (e) Average CG, CHG and CHH of hypo‐ and hyper‐CHH‐DMRs in the comparison of R0_vs_R2.

The DNA methylation patterns were explored throughout the protein‐coding genes (PCGs), transposon elements (TEs), TE‐related genes (TEGs) and their flanking 2‐kb regions between maternal Y668 (WT) and three generations (R0, R2 and R4). We observed that CG methylation showed a higher level in the gene body compare with TSS and TTS nearby regions and a similar methylation pattern (Figure 3a). Interestingly, the R0 plants showed higher DNA methylation levels than the WT and other regenerated plants (R2 and R4) in the PCGs (Figure 3a). Compared to the CG and CHG contexts, the difference in CHH methylation mainly occurred in 5′ upstream regions, which was called the CHH island (Gent *et al*., 2013). The DNA methylation patterns of TE sequences were compared in the CG, CHG and CHH contexts (Figure 3b). The CG, CHG and CHH methylation levels in TE regions were only moderately higher than in PCG regions. In addition, the CHH methylation level largely increased in R0 when compared with other plants. The CHH methylation level was moderately higher in TEGs than in PCGs (Figure 3c, Figure S1).

To further investigate DNA methylation changes, the differentially methylated regions (DMRs) were identified between WT and regenerated plants (Fisher's exact test, *FDR* < 0.05). We found that the number of the CHH‐DMRs (51 248, 52 209, 33 335) in each comparison group (WT_vs_R0, R0_vs_R2 and R2_vs_R4) was much larger than the number of CG‐DMRs (743, 813, 597) and CHG‐DMRs (971, 1081, 844) (Figure S2). The R0 plants had more hypermethylated CHH‐DMRs compared with WT plants, while the regenerated plants had more hypomethylated DMRs (Figure S3). Interestingly, the regenerated comparison groups (R0_vs_R2 and R2_vs_R4) had more overlapping CG‐ and CHG‐DMRs, while there were few overlapping CHH‐DMRs (Figure S4). The hypomethylated CHH‐DMRs (41 710) in WT plants had slightly higher CG and CHG methylation levels in R0 plants than WT plants, while hypermethylated CHH‐DMRs (9538) in WT showed no changes in their levels of CG and CHG methylation (Figure 3d). The whole CHH methylation level in CHH‐DMR was 10.19%, 28.86% in WT and R0 plants, respectively. The hypomethylated CHH‐DMRs (11 809) in R0 plants showed no changes in the CG and CHG contexts in regenerated plants, while the hypermethylated CHH‐DMRs (40 400) in R0 exhibited slightly higher CG and CHG methylation levels in R0 than in R2 (Figure 3e). For example, several hypermethylated CHH‐DMRs in R0 plants were shown from the R0 to R4 plants (Figure S5). These results suggested that the most CHH‐DMRs in WT and R0 plants were accompanied by CG and CHG methylation changes from WT to regenerated plants.

### DNA methylation patterns during the somatic embryogenesis of Jin668

**Figure 4:**
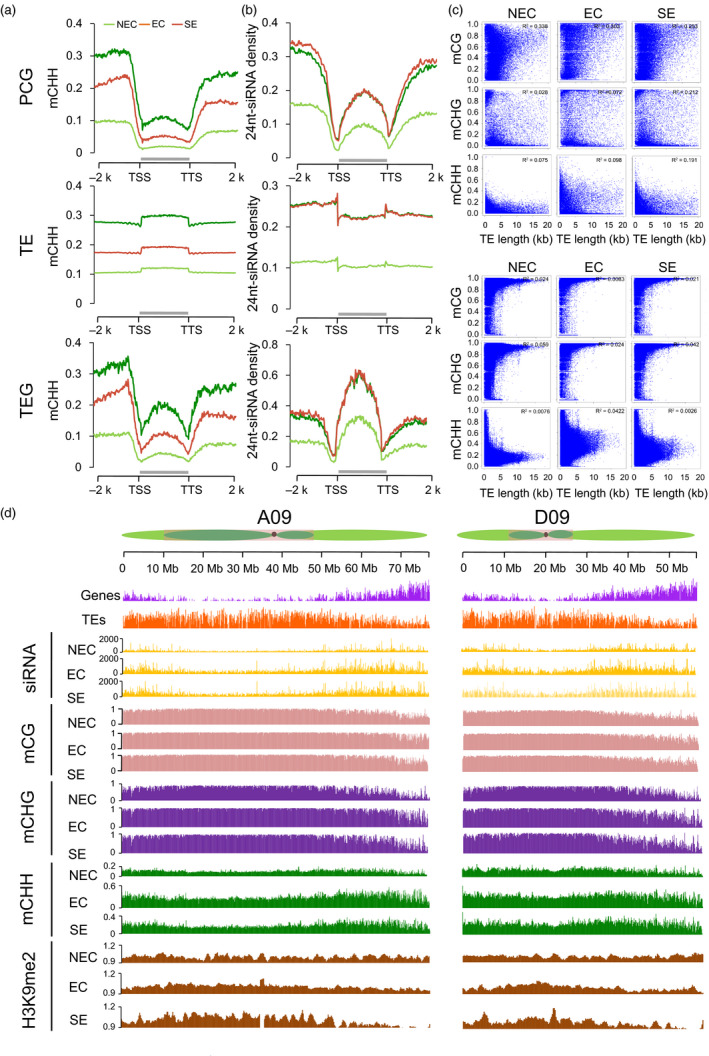
DNA methylation patterns in the callus of tissue culture process. (a‐b) Average CHH methylation levels and distribution of 24‐nt siRNAs in the PCGs, TEs and TEGs with their flanking 2‐kb regions. (c) Point scatter of the correlation between gene/TE lengths and DNA methylation levels in CG, CHG, and CHH contexts. (d) Distribution patterns of gene density, TE density, number of 24‐nt siRNAs, DNA methylation levels and H3K9me2 abundance in the A09 and D09 chromosomes in 100‐kb windows sliding 1‐kb. The heterochromatin (dark green), euchromatin (light green) and centromere regions (dark spot) of each chromosome are from Wang *et al*. (2015).

The relationship between the 24‐nt siRNAs’ abundance and DNA methylation was also investigated during the somatic embryogenesis. The data revealed that the 24‐nt siRNAs’ abundance was related to the CHH methylation in PCGs and TEGs (Figure 4a‐b). TEs occupy a large proportion (64.8%) of the *G. hirsutum* genome (Zhang *et al*., 2015). The different TE families revealed variable hypermethylation, but the EC and SE cells exhibited a much more difference than the NEC cells (Figure S6). Based on the length, all TEs were classified into long TEs (0.90%, >4‐kb), medium TEs (46.01%) and short TEs (53.99%, <0.5‐kb) (Wang *et al*., 2016a). The majority of long TEs were distributed in heterochromatic regions, and short TEs were distributed in euchromatic regions (Figure S7). Therefore, we focused on long TEs, which represent DNA methylation dynamics in heterochromatin. The long TEs were hypermethylated compared with short TEs in the CHH context (Figure S8). Furthermore, we also investigated the average methylation levels of CG, CHG and CHH in the different lengths of TEs and PCGs (Figure 4c). Long TEs were heavily methylated in the CG and CHG contexts and exhibited low methylation levels in the CHH context. These results indicated that the methylation levels in the CG/CHG contexts had similar distribution patterns in different lengths of TEs, while the CHH methylation levels were negatively methylated and CG/CHG were positively methylated with long TEs (Figure 4c). Furthermore, the CG methylation level was positively correlated with gene length (more than 0.25).

Non‐CG methylation primarily occurred in heterochromatin regions by the activity of the *CMT2*/*CMT3*‐involved pathway as described in previous studies (Gent *et al*., 2013; Lippman *et al*., 2004; Zhong *et al*., 2013). We firstly explored the dynamic DNA methylation that occurred during the somatic embryogenesis process through the RdDM and H3K9me2‐dependent pathways. To investigate the H3K9me2‐dependent pathway, we performed ChIP‐Seq on the H3K9me2 marker of NEC, EC and SE cells (Table S4). The PCGs, TEs, DNA methylation, 24‐nt siRNAs and H3K9me2 density were investigated in the *A*_*t*_‐ and *D*_*t*_‐subgenomes (Figure 4d). The genes and 24‐nt siRNAs were predominantly distributed in euchromatin regions, while the CG and CHG methylation and the enrichment of H3K9me2 modifications were preferentially enriched in heterochromatin regions, whereas the CHH methylation was found in euchromatin and heterochromatin regions (Figure 4d). The increased CHH methylation was consistent with the increase in the abundance of 24‐nt siRNAs from NEC to EC and is consistent with H3K9me2 pathway. The decreased CHH methylation from EC to SE was positive 24‐nt siRNAs’ enrichment and negative relative to H3K9me2 modification marks in euchromatic regions, but is not in heterochromatic regions (Figure 4d).

### The RdDM pathway contributes to the establishment of CHH methylation during the somatic embryogenesis process

**Figure 5:**
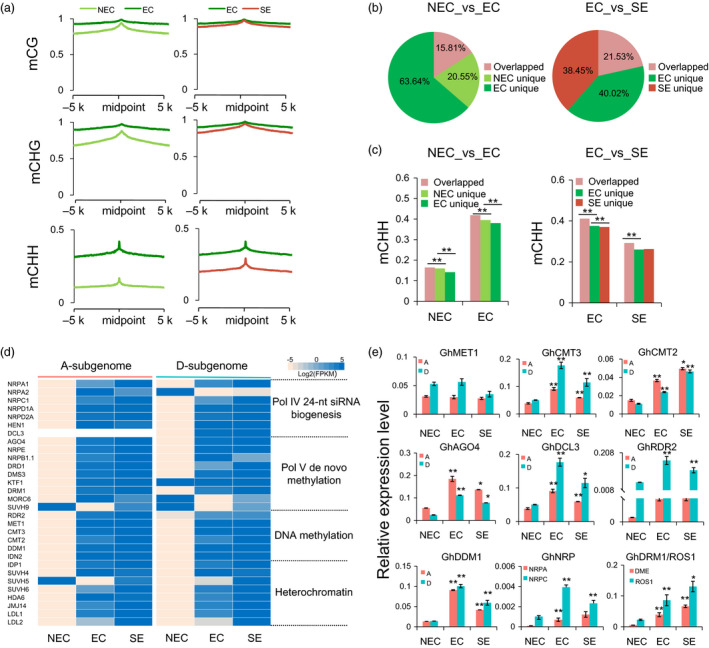
Active RNA‐directed DNA methylation during the tissue culture and somatic embryogenesis process. (a) The DNA methylation patterns of RdDM loci and their flanking upstream and downstream 5‐kb regions in NEC, EC and SE. (b) Pie chart showing proportions of overlapped and unique 24‐nt siRNAs during the continuous tissue culture process. (c) The CHH methylation patterns in cell‐specific and constitutive 24‐nt siRNAs in two groups, related to Fig. 5b. (d) The gene expression levels of RdDM pathway, demethylation and chromatin modification (left panel, *A*_*t*_‐subgenome; right panel, *D*_*t*_‐subgenome). The expression levels are normalized as Log2(FPKM) for each gene. (e) The expression patterns of RdDM components, DNA methyltransferases and de‐methyltransferases in NEC, EC and SE. *GhUbQ7* was used as the reference gene. Error bars indicate the standard error (S.E) of three biological replicates (Student's *t*‐test, *P <* *0.05, **P <* *0.01).

To examine the effects of the RdDM pathway on DNA methylation, the enrichment of 24‐nt siRNAs was analysed in gene body regions. These results showed that the number of genes with 24‐nt siRNAs’ production was less than that without 24‐nt siRNAs (Figure S9a). The DNA methylation levels of genes with overlapping 24‐nt siRNAs were much higher than those without overlapping 24‐nt siRNAs in the somatic embryogenesis process and of regenerated plants, especially in the CHG and CHH contexts (Figure S9b, Wilcoxon rank sum test, *P *<* *2.2e‐16). The effect of RdDM on the flanking 5‐kb regions was investigated in each sequence context. The DNA methylation levels peaked at the RdDM loci and decreased in the flanking 5‐kb regions, suggesting a true RdDM effect (Figure 5a). In addition, CHH methylation had much higher peaks than symmetric CG and CHG at RdDM loci. In EC and SE, methylation levels at the RdDM loci peaked sharply in the CHG and CHH contexts compared with the NEC stage. This result suggested that 24‐nt siRNA mapping regions could establish additional DNA methylation. We also compared the methylation levels of 24‐nt siRNA loci in different tissue culture stages. The different tissue cells had few common 24‐nt siRNA loci in the NEC_vs_EC and EC_vs_SE groups (Figure 5b). Interestingly, compared with specific 24‐nt siRNAs’ loci, the constitutive 24‐nt siRNA loci were hypermethylated in the CHH context in both groups (Figure 5c). Analysis of the RNA‐Seq data showed that the expression levels of genes encoding components of the canonical RdDM pathway (Matzke and Mosher, 2014) were increased in EC compared with the other cells and regenerated plants (Figure 5d and Data S1). Interestingly, *GhCMT3* and *GhCMT2* exhibited higher expression levels in EC than in NEC cells. Genes involved in the RdDM pathway, including *GhAGO4*,* GhDCL3*,* GhRDR2*,* GhNRPD*,* GhNRPA* and *GhNRPC,* were also up‐regulated from NEC to EC (Figure 5d, gene full name: Table S5). However, we found that the whole DNA methylation decreased from EC to SE, probably associated with the up‐regulated expression of genes encoding de‐methyltransferases (*ROS1*,* DME*) (Figure 5e). These results suggested that the RdDM pathway played a critical role in establishing and maintaining DNA methylation during somatic embryogenesis process.

### Tissue culture‐induced CHH hypermethylation in gene promoter regions contributes to functional gene expression during the somatic embryogenesis process

**Figure 6:**
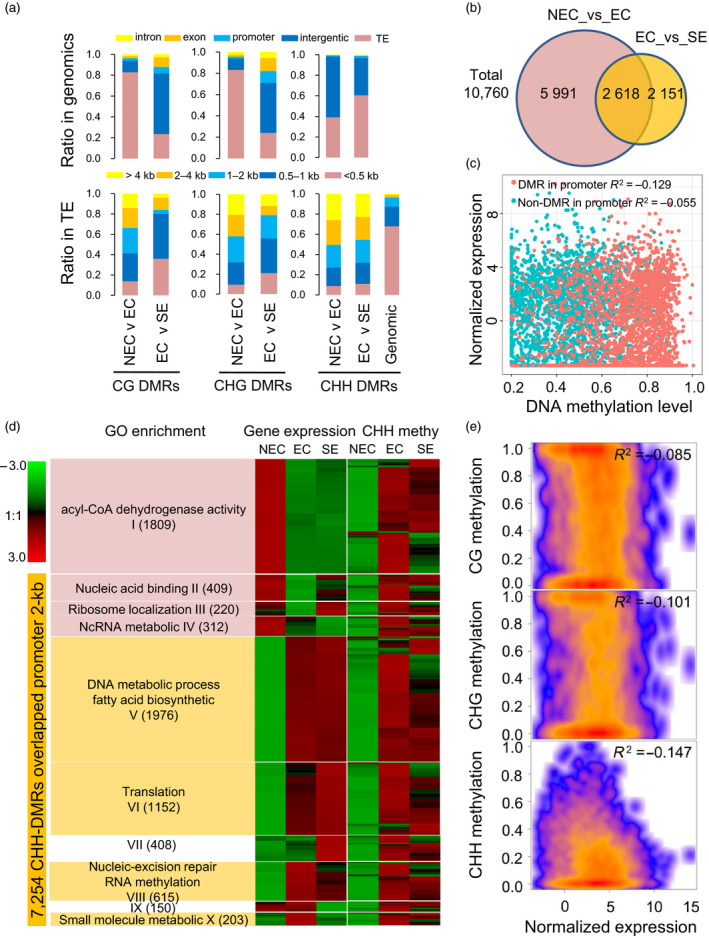
Tissue culture and somatic embryogenesis‐induced hypermethylation in ECs. (a) The genomic distribution of CG‐DMRs, CHG‐DMRs and CHH‐DMRs. The percentages of DMRs in intergenic regions, introns, exons, promoters and TEs (upper panel). The percentages of DMRs in TEs with different lengths (under panel). (b) The number of CHH‐DMRs overlapped promoters in the NEC_vs_EC and EC_vs_SE groups. (c) The expression correlation of CHH‐DMR overlapped genes and non‐CHH‐DMR overlapped genes (non‐DMR genes). (d) The relationship between the CHH methylation level and gene expression from the NEC to the EC stage. The gene expression patterns were categorized into 10 clusters (I‐X) according to the *K‐*means method. Each cluster was analysed with a GO enrichment analysis (*FDR* < 0.05). (e) The correlation between DNA methylation level in hypo‐CHH‐DMRs and gene expression level in the comparison of NEC_vs_EC. These CHH‐DMRs (2750) are overlapped with 2‐kb gene promoter regions.

The CG‐DMRs (2158–213), CHG‐DMRs (2171–179) and CHH‐DMRs (831 026–286 955) were identified in the NEC_vs_EC and EC_vs_SE comparison groups. These data showed that the hyper‐ and hypo‐DMRs were uniformly distributed in whole chromosomes (Figure S10). Interestingly, the NEC_vs_EC group had more overlapped CHH‐DMRs than the EC_vs_SE group, which was in contrast with the distribution patterns of the CG‐ and CHG‐DMRs (Figure S11). A large number of those DMRs overlapped with the gene body (exon, intron) and promoter 2‐kb regions, but more DMRs overlapped with the TE regions (Figure 6a). These DMRs were mainly enriched in long TEs relative to genomic distribution. In addition, DMRs in the EC_vs_SE comparison exhibited a higher abundance in short TEs relative to NEC_vs_EC, especially for CG‐ and CHG‐DMRs (Figure 6a).

In general, DNA methylation in gene promoter regions is thought to regulate gene transcription. In this study, we found that the relationship between gene expression and CG methylation levels in the promoter 2‐kb regions showed a weakly negative correlation (Table S6 and Figure S12). A total of 7254 CHH‐DMRs overlapped with promoters in the NEC_vs_EC and EC_vs_SE groups (Figure 6b). The 2618 common CHH‐DMRs were identified in two groups (Figure 6b), which DMR overlapped gene promoter of their gene expression exhibited higher correlation with the DNA methylation levels than non‐CHH‐DMR overlapped genes (Figure 6c). Expression patterns of these genes in the three stages (NEC, EC and SE) were divided into 10 clusters based on a *k‐means* method (Figure 6d). Genes in clusters I–IV were down‐regulated from the NEC to the EC stage, which corresponded to an increase in CHH methylation in the promoter 2‐kb regions. Strikingly, these genes were involved in acyl‐CoA dehydrogenase activity and the nucleic acid process (Fisher's exact test, *FDR* < 0.05). Genes in clusters V–VIII were enriched for the DNA metabolism and fatty acid biosynthetic processes, and their expression showed no significant correlation with DNA methylation levels. Furthermore, genes in clusters IX–X were down‐regulated from the EC to SE stage, accompanied by slight hypomethylation in the CHH context (Figure 6d). The overall gene expression levels (2750 down‐regulated genes from NEC to EC) exhibited a negative correlation with the CHH methylation level of the 2‐kb promoter, but it showed a weak correlation with the CG and CHG methylation levels (Pearson correlation coefficient, *R* ^2^ = −0.169, −0.112 and −0.084 in CG, CHG and CHH, respectively, *P *<* *2.2e‐07, Figure 6e; Data S2).

### The increased CHH methylation in promoter regions resulted in some hormone‐related genes down‐regulation from the NEC to the EC stage

**Figure 7:**
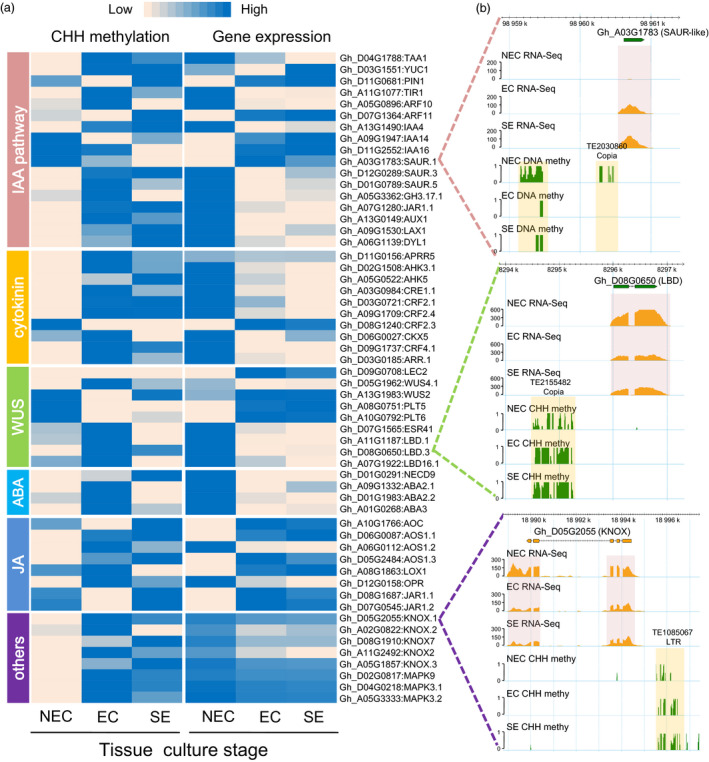
CHH methylation regulates the expression of functional genes during the tissue culture process. (a) The heatmap shows expression patterns and CHH methylation levels of 2‐kb promoters for IAA, cytokinin, WUS, ABA and JA‐related genes and other functional genes. (b) The DNA methylation patterns and gene expression levels are shown by the genome browser snapshots in the tissue culture process. The orange box shows two CHH‐DMRs. The red box shows the RNA‐Seq data.

Dynamic DNA methylation patterns play an important role in regulating dedifferentiation and redifferentiation in tissue culture process and act via the hormone signalling pathway (Ikeuchi *et al*., 2016; Miguel and Marum, 2011). The tissue culture process elaborates a complex network of interactions among plant growth regulators, mainly including auxins, cytokinins, abscisic acids (ABA), jasmonic acid (JA) and *WUSCHEL*‐related homeobox (*WUS*) in the development of somatic embryos (De‐la‐Pena *et al*., 2015). We analysed DEGs that overlapped with CHH‐DMRs in the NEC_vs_EC and EC_vs_SE groups. In this study, 57 genes (including 18 IAA‐related, 10 cytokinin‐related, 9 *WUSCHEL*‐related, 4 ABA‐related, 8 JA‐related and 8 marker genes) were differentially expressed during the tissue culture process, which might be associated with differential CHH methylation levels in the 2‐kb promoter (Figure 7a and Data S3). Expression patterns and CHH methylation levels of 32 genes related to auxin synthesis, transport and signalling were investigated from NEC to EC. The *PIN1*,* SAUR.1*,* IAA14* and *IAA16* were hypermethylated in NEC cells, but their expression was repressed from the NEC to the EC stage (full gene names: Data S3). Notably, the expression levels of most genes (14) were down‐regulated from the NEC to the EC stage, with the increased CHH methylation level, such as *ARFs*,* GH3*,* SAUR‐like*,* JARs*,* AUX1*,* LAX1*,* DYL1* and *IAA4/29* (Figure 7a). For example, a hypermethylated DMR was detected for a SAUR‐like auxin‐responsive gene (Gh_A03G1783) at the NEC cell, when it displayed a lower expression level than in the EC and SE stages (Figure 7b). For example, the Gh_A03G1783 contains a short TE (*Copia*) in the promoter 2‐kb region. The methylation level in the flanking *Copia* of Gh_A03G1783 decreased from NEC to EC. These data indicated that additional CHH methylation in the form NEC to EC stage may be correlated with decreased expression of IAA‐related genes during the tissue culture process.

Cytokinin biosynthesis and signalling have been implicated in many aspects of plant development, including cell division, shoot initiation and embryonic development in *Arabidopsis* (Kieber and Schaller, 2014). We next explored DNA methylation patterns and the expression of cytokinin signalling pathway‐related genes. Ten genes exhibited hypermethylation in the 2‐kb promoter regions of EC cells, and these genes were silenced from NEC to EC stage (Figure 7a and Data S3). Strikingly, two CRF2 genes were hypermethylated in NEC, accompanied by down‐regulated expression from NEC to EC stage. The *WUSCHEL* and WUS‐related homeobox (*WOX*) transcription factors are the key switches of stem cell differentiation in shoot apical meristem (SAM), root apical meristem (RAM) and callus cells (Sarkar *et al*., 2007). Promoters of the *LEC2*,* WUS4.1*,* PLT5/6* and *ESR12* genes were slightly hypermethylated in NEC, while other WUS‐related gene promoters (*LBD* and *ESR*) were hypomethylated in NEC (Figure 7a). The CHH methylation level of the 2‐kb promoter region of the Lateral organ boundaries domain family gene (*LBD*, Gh_D08G0650) gradually increased from the NEC to the EC stage, which might be associated with its down‐regulated expression (Figure 7b).

In addition to auxin‐ and cytokinin‐related genes, the expression levels of other plant hormone signalling‐related genes and tissue culture marked genes were negatively correlated with the CHH methylation levels of their 2‐kb promoter regions (Figure 7a). The *KNOX* family transcription factor (Gh_D05G2055), essential to maintain callus cell fate *in vitro* (Abarca and Diaz‐Sala, 2009), was down‐regulated from the NEC to the EC stage, which might be associated with the hypermethylation of its promoter‐inserted LTR transposon in all three cells (Figure 7b).

### The inhibition of DNA methylation in callus activated some hormone‐related genes transcription and may promote the somatic embryogenesis

**Figure 8:**
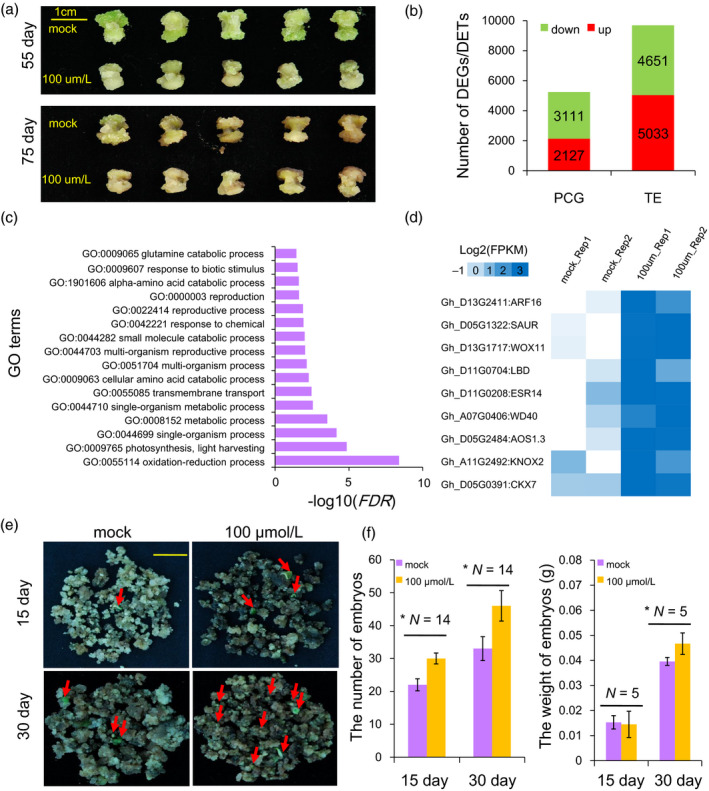
Gene and TE expression in cotton callus and somatic embryos under zebularine treatment. (a) The phenotypes of zebularine‐treated cotton embryogenic calluses for 55 and 75 day. (b) Identification of differentially expressed genes and TEs in cotton calluses after zebularine treatment. (c) GO enrichment analysis of DEGs following zebularine treatment of cotton callus. (d) The expression patterns of tissue culture marker genes in the mock and zebularine‐treated calluses (corresponding to Figure 7a). (e) Embryogenesis of calluses cultured on medium with 0, and 100 μmol/L of zebularine for 15 and 30 days. (f) The number of embryos and weight under the zebularine treatment calluses.

To further examine the effects of DNA methylation on the callus redifferentiation, the hypocotyl‐derived NEC of the cotton Jin668 was treated with 100 μmol/L concentrations of zebularine in the subculture medium. After successive culture for two cycles (1 month for one cycle), the callus became deep green (Figure 8a). To investigate the possible effects of zebularine on callus development, a transcriptome analysis was performed in mock and zebularine‐treated callus (for 75 days) (Table S7). Analysis of the transcription of DNA methyltransferases by RNA‐Seq showed that most genes had no significant changes, but, only GhMET1 was down‐regulated under zebularine treatment (Table S8). This result might indicate the whole DNA methylation was decreased in NEC under zebularine treatment. At the gene level, we found that a total of 5238 genes exhibited differential expression (Figure 8b). In addition, RNA‐Seq data also showed that the number of up‐regulated TEs was larger than down‐regulated TEs (5031 vs 4651, chi‐squared test, *P *<* *0.001) (Figure 8b). GO enrichment analysis showed that these differentially expressed genes (DEGs) were involved in the oxidation–reduction process, stress and development, among others (Figure 8c, Fisher's exact test, *FDR* < 0.001). The expression of hormone‐related and tissue culture marker genes (corresponding to Figure 7, total 57 genes) was analysed. The result showed that six genes were activated under zebularine treatment, including *IAA14*,* CKX6*,* LBD1/3*,* LOX1* and *CRF4*.1 (Figure 8d).

The redifferentiation process (from the EC to the SE stage) was also examined after zebularine treatment. After the embryogenic callus was cultured on medium containing mock and 100 μmol/L for 15 and 30 days, the number of total somatic embryos of 100 μmol/L treatments were significantly increased compared to the mock for 15 and 30 days, and the weight of embryos was increased for 30 days (Figure 8e‐f). We observed an increasingly positive effect on somatic embryogenesis with the zebularine treatment, suggesting that the inhibition of DNA methylation might play a positive role in regulating cotton somatic embryogenesis.

## Discussion

Extensive functional genomics studies revealed cell differentiation in shoots and roots’ apical meristem (Ikeuchi *et al*., 2015). Previous studies have investigated histone modifications and siRNA levels in plant cell suspension cultures *in vitro* (Tanurdzic *et al*., 2008). Genomewide DNA methylation analysis in leaves and leaf‐derived callus exhibited a differential methylation in tissue culture (Vining *et al*., 2013; Zakrzewski *et al*., 2017). In this study, we revealed the effects of the epigenome on the cotton tissue culture process in the callus, somatic embryos and several regenerated plants through DNA methylation, RNA‐Seq, sRNA‐Seq and ChIP‐Seq analyses. We observed that an increase in CHH methylation predominantly occurred in EC cells, accompanied by an enrichment in the 24‐nt siRNAs and H3K9me2. Interestingly, CHH methylation and 24‐nt siRNAs were significantly eliminated in regenerated plants (from R0 to R4), suggesting that CHH methylation might be closely linked to the enrichment of 24‐nt siRNAs in the whole genome. We found the differences in the CG and CHG methylation in different tissue culture cells and regenerated plants, which is consistent with data from somatic tissues in rice and *Arabidopsis* regenerated plants (Sarkar *et al*., 2007; Stroud *et al*., 2013), both of which showed more variation in CHH methylation during different tissue culture process.

Tissue culture is the major methodology used for the production of transgenic crops and clonally propagated plants, which often generates somaclonal variation in the callus as well as the regenerated plants. For regenerated plants, the DNA methylation profile is quite different in variant plant species. Studies on the methylation patterns in rice and maize have revealed that methylation‐lost loci are more common than methylation‐gained loci after tissue culture and somatic embryogenesis (Stelpflug *et al*., 2014; Stroud *et al*., 2013). Analysis expression patterns of *MET*,* CMT* and *DRM* methyltransferases exhibited higher expression levels in fast‐growing calli and then hypermethylated in regenerated plants (Rival *et al*., 2008). Somaclonal variation has led to diverse and unexpected phenotypes in regenerated oil palm plants from tissue culture, which drastically reduced the yield because of DNA methylation changes in the *LINE* retrotransposon (Ong‐Abdullah *et al*., 2015). In contrast to rice regenerated plants, we did not observe global hypomethylation regenerated cotton plants. Instead, we found that certain genomic sites had gained DNA methylation during the R0 plants after genetic transformation and plant regeneration, which were consistent with previous reports regarding regenerated banana plants (Peraza‐Echeverria *et al*., 2001). CG and CHG methylation were found to disappear in specific regions of the regenerated plants (R2, R4) compared with the R0 plants. Compared with early regenerated lines (R0, R2), the elite “Jin668” was hypomethylated in the CHH context and we speculate that the Jin668 plants could have a higher regenerative ability due to hypomethylation that occurred through the SRA strategy. Our methodology may be applied to improve the genetic transformation and plant regeneration efficiency in a range of recalcitrant plant species.

Several studies have shown that the cells and somatic embryos contain enormous amounts of differentially expressed genes during somatic embryogenesis in cotton (Yang *et al*., 2012, 2013). Plant hormones such as auxin obviously affect the somatic embryogenesis process, but how gene expression is regulated at the epigenetic level is still not clear. In this study, we analysed the CHH methylation levels of plant hormone‐related genes and their genomewide expression profiles in NEC, EC and SE.

In plants, CHH methylation is mediated by both RdDM and CMT2‐involved pathways requiring H3K9me2 (Du *et al*., 2015). The RdDM loci are mainly located in euchromatic regions, and the *CMT2*‐involved pathway often acts in heterochromatic regions (Zemach *et al*., 2013). Interestingly, the DNA methylation levels were significantly higher in 24‐nt siRNAs mapping genes than nonmapping genes. The increase in non‐CG methylation levels in 24‐nt marked genes might be caused by activation of the RdDM pathway during plant tissue culture and regenerated plants. To support this hypothesis, we comprehensively investigated the expression patterns of genes involved in the RdDM pathway such as *NRPA*,* NRPC*,* NRPD*,* RDR2* and *DCL3* (Matzke and Mosher, 2014). Our results suggested that these genes were up‐regulated from the NEC to the EC stage, but exhibited no changes from the EC to the SE stage. Nevertheless, the demethylation enzymes, including *ROS1* and *DME*, were up‐regulated from EC to SE, which suggested a possibility of active demethylation. The increase in H3K9me2 deposition was probably associated with the increase in CHH methylation from NEC to EC. However, the level of H3K9me2 deposition was decreased from EC to SE. In addition, the expression of *CMT2* was up‐regulated from the EC to the SE stage. These suggest that the RdDM and H3K9me2‐dependent pathways may contribute to CHH methylation dynamics synergistically from NEC to EC stages. Taken together, these findings will enhance our understanding of the role of the *CMT2*‐involved pathway and the RdDM pathway in the maintenance of DNA methylation from NEC to EC.

## Experimental procedures

### Plant materials

Upland cotton (*Gossypium hirsutum* L.) strict inbred Y668 (from the Henan Academy of Agriculture Science, China) was used as the maternal genotype for developing Jin668. The *Agrobacterium* strain EHA105, harbouring the pBIN m‐gfp5‐ER plasmid, was used for transformation. *GFP* fluorescence detection was performed following our previous report (Jin *et al*., 2012). The tissue culture and *Agrobacterium‐*mediated genetic transformation process for the development of Jin668 were performed following our previous publications (Jin *et al*., 2012; Tian *et al*., 2015; Wang *et al*., 2016b). In brief, the hypocotyls of maternal inbred Y668 cotton plantlets were used as explants for *Agrobacterium‐*mediated genetic transformation with *GFP* as a reporter gene. The seeds from regenerated plants (R0) were screened by PCR and *GFP* fluorescence detection. The negative plants (null, R0 progeny) were selected for further use. All the null (R0‐null) plants were strictly self‐fertilized and generated the seeds for the second‐round tissue culture (no *GFP*) and somatic embryogenesis to generate the R1 generation. Then, this regeneration process was repeated thrice to generate the R2, R3 and R4 inbred line generations. The R4 generation was then defined as Jin668 (widely used for the cotton genetic transformation in our laboratory now), and this whole process was defined as the Successive Regeneration Acclimation (SRA) strategy. For the comparison of regeneration ability from different generations in SRA process, the regeneration ability was calculated by the number of explants producing embryogenic callus per 100 explants (hypocotyl segments) within 4‐month tissue culture process and the experiment was repeated for three times (Student's *t*‐test was applied for the data analysis).

### Zebularine treatment during the tissue culture of Jin668

The tissue culture process was described previously with minor modifications (Jin *et al*., 2006a). Seeds of Jin668 were decoated, surface‐sterilized and transferred to 1/2 Murashige and Skoog (MS) medium for culture at 28 °C in the dark for 7 days. The hypocotyls of the seedlings were cut into 5–7‐mm segments. Callus induction was carried out on callus‐inducing medium (Jin *et al*., 2006a) with 100 μmol/L concentrations of zebularine treatment (Selleck, S7113). Different callus tissues from different stages during cotton somatic embryogenesis were sampled as described previously (Yang *et al*., 2012).

### Bisulphite‐treated sequencing (BS‐Seq) library construction

The NEC cells at 75 days, EC, and SE collected from different Petri dishes of Jin668 were pooled (each type have pooled six samples) and immediately frozen in liquid nitrogen (Figure 1d). Genomic DNA was extracted from NEC, EC, SE and leaves using the Plant Genomic DNA kit (TIANGEN, Cat. #DP305‐03). As well, the leaves of Y668 (WT) and the leaves of R0, R2, R4 regenerated offspring plants were extracted Genomic DNA. First, 2 μg of high‐quality genomic DNA was fragmented between 300 and 500 bp by sonication. Then, Illumina adapters were ligated following the manufacturer's protocols, after which fragments with adapters were treated with bisulphite using the EZ DNA Methylation‐Gold™ Kit (Catalog No. D5005). At the same time, unmethylated lambda DNA (Promega) was treated as a control. Finally, the treated DNA fragments were amplified and cleaned up with AMPure Beads. The paired‐end sequencing of the BS‐Seq library was performed on the Illumina HiSeq 3000 platform.

### BS‐Seq data analysis

The BS‐Seq raw reads were preprocessed with Trimmomatic (version 0.32) software to remove low‐quality reads and adapters. The clean reads were mapped to the genome of *G. hirsutum* acc. TM‐1 (Zhang *et al*., 2015) using bismark (version 0.13.0, ‐N 1 ‐L 30) software (Krueger and Andrews, 2011). Then, methylated cytosines were called from the uniquely mapped reads using the bismark methylation extractor under standard parameters. The bisulphite nonconversion rate (0.006) was calculated by sequencing the bisulphite‐converted lambda DNA. Methylated cytosines covered by at least three reads were identified with a binomial distribution test (*P *<* *1e‐05) and methylated ratio >25%. The CG, CHG and CHH methylation levels at flanking 2‐kb regions and gene/TE body were calculated based on the average methylation level of a 100 interval using customized Perl scripts. Differentially methylated regions (DMR) were identified using the Fisher's exact test (Benjamini–Hochberg‐corrected *FDR* < 0.05) in 100 bp bins across the genome. Finally, only bins that contained 10 informative cytosines (*i.e*. covered by >3 reads) in any two samples were considered as DMRs. The differentially methylated cytosines (DMCs) in any two samples were defined by a binomial test. We required an absolute methylation difference of 0.7, 0.5 and 0.1 for CG, CHG and CHH methylation in any two samples to designate DMCs (Stroud *et al*., 2013).

### RNA sequencing (RNA‐Seq) and data analysis

Total RNA was isolated from different stages of embryogenic calluses of Jin668 and leaves of Y668 (WT), R0, R2 and R4 using a modified guanidine thiocyanate method (Tu *et al*., 2007). RNA‐Seq libraries were constructed using the Illumina TruSeq Stranded RNA kit (San Diego, CA) following the manufacturer's recommendations with two biological replicates. After removing low‐quality reads, clean reads were mapped to the *G. hirsutum* genome by Tophat2 (version 2.0.13) with the default parameters (Trapnell *et al*., 2010). Gene expression levels were calculated as fragments per kilobase per million (FPKM) by Cufflinks (Trapnell *et al*., 2012). The Cuffdiff was run to identify differentially expressed genes (DEGs) (*P *<* *0.05 and log2 |ratio| ≥ 2) (Trapnell *et al*., 2012). Gene ontology (GO) enrichment was analysed by the Blast2GO procedure (Fisher's exact test, *P *<* *0.05).

### Small RNA sequencing (sRNA‐Seq) and data analysis

The sRNA‐Seq libraries were constructed from the same tissues as the mRNA libraries using the Illumina TruSeq Small RNA Sample Preparation protocol and sequenced (HiSeq 2000, single‐end 50‐bp reads) with two biological replicates. After sequencing, the raw reads were trimmed with the NGSQC toolkit to remove low‐quality reads and adaptors (Patel and Jain, 2012). Then, these reads were mapped to the Rfam database to exclude snRNA, snoRNA, tRNA and rRNA reads. The final clean reads were mapped to the *G. hirsutum* genome using bowtie (‐v 0 ‐m 200). Structure‐ and probability‐based methods were adopted to predict miRNAs as described previously (Paterson *et al*., 2012). The remaining unique reads were subjected to further analysis.

### Chromatin immunoprecipitation (ChIP) experiment and data analysis

ChIP was performed as described previously (Sun and Zhou, 2008), with minor modifications. Chromatin was isolated from 1 g of cotton embryogenic callus of Jin668. Chromatin was sonicated to 200–300 bp fragments for 30 min with a 30 s on and 30 s off cycle on ice, except for the SE sample, which had a total of 20 min of sonication. After sonication, 4.5 μL antibody H3K9me2 (Abcam: ab1220, Shanghai, China) was cross‐linked with 50 μL protein A Dyna beads at 4 °C in a mute mixer overnight and added to 20 μL chromatin fragment supernatant for chromatin immunoprecipitation. For each sample, 20 μL of supernatant was kept as the input control. After washing and elution, products were de‐cross‐linked for 6 h at 65 °C. Finally, products were purified with 2 μL proteinase K, 10 μL EDTA, 5 μL RNase A and 20 μL Tris–HCl for 1 h at 45 °C. Purification products were checked using the PicoGreen assay (Invitrogen Q‐bit) and Agilent BioAnalyzer DNA 100 chip (Agilent, CA) for constructing Illumina sequencing libraries following the manufacturer's recommendations (Illumina HiSeq 3000).

After removing low‐quality reads, the clean reads were mapped to the *G. hirsutum* genome by Bowtie2 (version 2.2.4) using the default parameters. MACS software was used to identify histone modification peaks (*P *<* *0.001) (Zhang *et al*., 2008).

### Quantitative RT‐PCR (qRT‐PCR)

Quantitative RT‐PCR was performed as previously described to confirm the RNA‐Seq data (Li *et al*., 2016). The relative expression level of each gene was normalized using the cotton Ubiquitin 7 (*GhUb7*) gene as a reference gene.

### Accession numbers

The raw reads of BS‐Seq, strand‐specific RNA‐Seq, sRNA‐Seq and ChIP‐Seq data have been submitted to the NCBI Sequence Read Archive under the Bioproject ID: PRJNA380842.

## Conflict of interest

The authors have declared that no competing interests exist.

## Supporting information


**Figure S1** CG, CHG and CHH methylation patterns of TEGs and PCGs.
**Figure S2** The numbers of hypomethylated (Hypo) and hypermethylated (Hyper) context‐specific differentially methylated regions (DMRs) in regenerated plants.
**Figure S3** A circos plot of the TE density, gene density, CG‐differentially methylated region (DMR), CHG‐DMR, and CHH‐DMR in comparisons of WT_vs_R0, R0_vs_R2 and R2_vs_R4. The hyper‐DMRs and hypo‐DMRs were normalized by the density. The red and green colors represent hyper‐DMRs and hypoDMRs, respectively.
**Figure S4** The number of overlapped CG‐, CHG‐ and CHH‐DMRs in the three groups.
**Figure S5** An example of hypermethylation in the comparison of R0_vs_R2. Every orange triangle represents one DMR.
**Figure S6** CHH methylation level in different TE families.
**Figure S7** The distribution of TEs length. The upper panel are short TE (<0.5‐kb), middle TE (0.5‐4‐kb), and long TE (>4‐kb) according to TE length. The long TE divide into long *Gypsy*, long *Copia* and the others (under panel).
**Figure S8** Average distribution of CHH methylation in short TEs and long TEs with their flanking 2‐kb regions.
**Figure S9** The RdDM effects in gene body region during the somatic embryogenesis process.
**Figure S10** The circos plot shows the TE density, gene density, CG‐DMR, CHGDMR and CHH‐DMR in NEC_vs_EC, EC_vs_SE and SE_vs_R0 groups.
**Figure S11** The numbers of overlapped CG‐, CHG‐, CHH‐DMRs during tissue culture development stages and from WT to regeneration plants.
**Figure S12** Gene expression have weak negatively correlation with DNA methyl of promoter 2‐kb.

**Table S1** Summary of BS‐Seq data in *G. hirsutum*.
**Table S2** Identification of methylated cytosines in each of the seven samples.
**Table S3** Summary of small RNA‐Seq data.
**Table S4** Summary of H3K9me2 data.
**Table S5** Summary of the full names of DNA methylation pathway related genes.
**Table S6** Summary of RNA‐Seq data.
**Table S7** Summary of RNA‐Seq data from mock and zebularine treatment of callus.
**Table S8** The expression patterns of RdDM related genes and DNA methyltransferases under zebularine treatment.

**Data S1** The expression and mCHH methylation encoding RdDM pathway genes.

**Data S2** The hyper‐DMRs (promoter 2‐kb) in ECs and corresponding gene expression from NEC to EC.

**Data S3** The expression and mCHH methylation hormone‐related genes somatic embryogenesis process.

## References

[pbi12988-bib-0001] Abarca, D. and Diaz‐Sala, C. (2009) Reprogramming adult cells during organ regeneration in forest species. Plant Signal. Behav. 4, 793–795. 19820297 10.4161/psb.4.8.9238 PMC2801403

[pbi12988-bib-0002] Chen, Z.J. (2013) Genomic and epigenetic insights into the molecular bases of heterosis. Nat. Rev. Genet. 14, 471–482. 23752794 10.1038/nrg3503

[pbi12988-bib-0003] De‐la‐Pena, C. , Nic‐Can, G.I. , Galaz‐Avalos, R.M. , Avilez‐Montalvo, R. and Loyola‐Vargas, V.M. (2015) The role of chromatin modifications in somatic embryogenesis in plants. Front. Plant. Sci. 6, 635. 26347757 10.3389/fpls.2015.00635 PMC4539545

[pbi12988-bib-0004] Du, J. , Zhong, X. , Bernatavichute, Y.V. , Stroud, H. , Feng, S. , Caro, E. , Vashisht, A.A. *et al*. (2012) Dual binding of chromomethylase domains to H3K9me2‐containing nucleosomes directs DNA methylation in plants. Cell, 151, 167–180. 23021223 10.1016/j.cell.2012.07.034 PMC3471781

[pbi12988-bib-0005] Du, J. , Johnson, L.M. , Jacobsen, S.E. and Patel, D.J. (2015) DNA methylation pathways and their crosstalk with histone methylation. Nat. Rev. Mol. Cell Biol. 16, 519–532. 26296162 10.1038/nrm4043 PMC4672940

[pbi12988-bib-0006] Fukai, E. , Umehara, Y. , Sato, S. , Endo, M. , Kouchi, H. , Hayashi, M. , Stougaard, J. *et al*. (2010) Derepression of the plant Chromovirus LORE1 induces germline transposition in regenerated plants. PLoS Genet. 6, e1000868. 20221264 10.1371/journal.pgen.1000868 PMC2832683

[pbi12988-bib-0007] Gehring, M. , Huh, J.H. , Hsieh, T.F. , Penterman, J. , Choi, Y. , Harada, J.J. , Goldberg, R.B. *et al*. (2006) DEMETER DNA glycosylase establishes MEDEA polycomb gene self‐imprinting by allele‐specific demethylation. Cell, 124, 495–506. 16469697 10.1016/j.cell.2005.12.034 PMC4106368

[pbi12988-bib-0008] Gent, J.I. , Ellis, N.A. , Guo, L. , Harkess, A.E. , Yao, Y. , Zhang, X. and Dawe, R.K. (2013) CHH islands: de novo DNA methylation in near‐gene chromatin regulation in maize. Genome Res. 23, 628–637. 23269663 10.1101/gr.146985.112 PMC3613580

[pbi12988-bib-0009] Gong, Z. , Morales‐Ruiz, T. , Ariza, R.R. , Roldan‐Arjona, T. , David, L. and Zhu, J.K. (2002) ROS1, a repressor of transcriptional gene silencing in Arabidopsis, encodes a DNA glycosylase/lyase. Cell, 111, 803–814. 12526807 10.1016/s0092-8674(02)01133-9

[pbi12988-bib-0010] Hatorangan, M.R. , Laenen, B. , Steige, K. , Slotte, T. and Kohler, C. (2016) Rapid evolution of genomic imprinting in two species of the brassicaceae. Plant Cell, 28, 1815–1827. 27465027 10.1105/tpc.16.00304 PMC5006707

[pbi12988-bib-0011] Henderson, I.R. and Jacobsen, S.E. (2007) Epigenetic inheritance in plants. Nature, 447, 418–424. 17522675 10.1038/nature05917

[pbi12988-bib-0012] Ikeuchi, M. , Iwase, A. and Sugimoto, K. (2015) Control of plant cell differentiation by histone modification and DNA methylation. Curr. Opin. Plant Bio. 28, 60–67. 26454697 10.1016/j.pbi.2015.09.004

[pbi12988-bib-0013] Ikeuchi, M. , Ogawa, Y. , Iwase, A. and Sugimoto, K. (2016) Plant regeneration: cellular origins and molecular mechanisms. Development, 143, 1442–1451. 27143753 10.1242/dev.134668

[pbi12988-bib-0014] Jin, S. , Liang, S. , Zhang, X. , Nie, Y. and Guo, X. (2006a) An efficient grafting system for transgenic plant recovery in cotton (Gossypium hirsutum L.). Plant Cell, Tissue Organ Cult. 85, 181–185.

[pbi12988-bib-0015] Jin, S. , Zhang, X. , Nie, Y. , Guo, X. , Liang, S. and Zhu, H. (2006b) Identification of a novel elite genotype for in vitro culture and genetic transformation of cotton. Biol. Plantarum. 50, 519–524.

[pbi12988-bib-0016] Jin, S. , Liu, G. , Zhu, H. , Yang, X. and Zhang, X. (2012) Transformation of Upland Cotton (Gossypium hirsutum L.) with gfp Gene as a Visual Marker. J. Integr. Agr. 11, 910–919.

[pbi12988-bib-0017] Kieber, J.J. and Schaller, G.E. (2014) Cytokinins. Arabidopsis Book, 12, e0168. 24465173 10.1199/tab.0168 PMC3894907

[pbi12988-bib-0018] Krueger, F. and Andrews, S.R. (2011) Bismark: a flexible aligner and methylation caller for Bisulfite‐Seq applications. Bioinformatics, 27, 1571–1572. 21493656 10.1093/bioinformatics/btr167 PMC3102221

[pbi12988-bib-0019] Law, J.A. and Jacobsen, S.E. (2010) Establishing, maintaining and modifying DNA methylation patterns in plants and animals. Nat. Rev. Genet. 11, 204–220. 20142834 10.1038/nrg2719 PMC3034103

[pbi12988-bib-0020] Leelavathi, S. , Sunnichan, V.G. , Kumria, R. , Vijaykanth, G.P. , Bhatnagar, R.K. and Reddy, V.S. (2004) A simple and rapid Agrobacterium‐mediated transformation protocol for cotton (Gossypium hirsutum L.): embryogenic calli as a source to generate large numbers of transgenic plants. Plant Cell Rep. 22, 465–470. 13680138 10.1007/s00299-003-0710-x

[pbi12988-bib-0021] Li, J. , Zhu, L. , Hull, J.J. , Liang, S. , Daniell, H. , Jin, S. and Zhang, X. (2016) Transcriptome analysis reveals a comprehensive insect resistance response mechanism in cotton to infestation by the phloem feeding insect Bemisia tabaci (whitefly). Plant Biotechnol. J. 14, 1956–1975. 26923339 10.1111/pbi.12554 PMC5042180

[pbi12988-bib-0022] Lippman, Z. , Gendrel, A.V. , Black, M. , Vaughn, M.W. , Dedhia, N. , McCombie, W.R. , Lavine, K. *et al*. (2004) Role of transposable elements in heterochromatin and epigenetic control. Nature, 430, 471–476. 15269773 10.1038/nature02651

[pbi12988-bib-0023] Luo, J. , Liang, S. , Li, J. , Xu, Z. , Li, L. , Zhu, B. , Li, Z. *et al*. (2017) A transgenic strategy for controlling plant bugs (Adelphocoris suturalis) through expression of double‐stranded RNA homologous to fatty acyl‐coenzyme A reductase in cotton. New Phytol. 215, 1173–1185. 28608990 10.1111/nph.14636

[pbi12988-bib-0024] Matzke, M.A. and Mosher, R.A. (2014) RNA‐directed DNA methylation: an epigenetic pathway of increasing complexity. Nat. Rev. Genet. 15, 394–408. 24805120 10.1038/nrg3683

[pbi12988-bib-0025] Miguel, C. and Marum, L. (2011) An epigenetic view of plant cells cultured in vitro: somaclonal variation and beyond. J. Exp. Bot. 62, 3713–3725. 21617249 10.1093/jxb/err155

[pbi12988-bib-0026] Mosher, R.A. and Melnyk, C.W. (2010) siRNAs and DNA methylation: seedy epigenetics. Trends Plant Sci. 15, 204–210. 20129810 10.1016/j.tplants.2010.01.002

[pbi12988-bib-0027] Ong‐Abdullah, M. , Ordway, J.M. , Jiang, N. , Ooi, S.E. , Kok, S.Y. , Sarpan, N. , Azimi, N. *et al*. (2015) Loss of Karma transposon methylation underlies the mantled somaclonal variant of oil palm. Nature, 525, 533–537. 26352475 10.1038/nature15365 PMC4857894

[pbi12988-bib-0028] Patel, R.K. and Jain, M. (2012) NGS QC toolkit: a toolkit for quality control of next generation sequencing data. PLoS ONE, 7, e30619. 22312429 10.1371/journal.pone.0030619 PMC3270013

[pbi12988-bib-0029] Paterson, A.H. , Wendel, J.F. , Gundlach, H. , Guo, H. , Jenkins, J. , Jin, D. , Llewellyn, D. *et al*. (2012) Repeated polyploidization of Gossypium genomes and the evolution of spinnable cotton fibres. Nature, 492, 423–427. 23257886 10.1038/nature11798

[pbi12988-bib-0030] Peraza‐Echeverria, S. , Herrera‐Valencia, V.A. and Kay, A. (2001) Detection of DNA methylation changes in micropropagated banana plants using methylation‐sensitive amplification polymorphism (MSAP). Plant Sci. 161, 359–367. 11448766 10.1016/s0168-9452(01)00421-6

[pbi12988-bib-0031] Rival, A. , Jaligot, E. , Beule, T. and Finnegan, E.J. (2008) Isolation and expression analysis of genes encoding MET, CMT, and DRM methyltransferases in oil palm (Elaeis guineensis Jacq.) in relation to the ‘mantled’ somaclonal variation. J. Exp. Bot. 59, 3271–3281. 18640997 10.1093/jxb/ern178

[pbi12988-bib-0032] Sarkar, A.K. , Luijten, M. , Miyashima, S. , Lenhard, M. , Hashimoto, T. , Nakajima, K. , Scheres, B. *et al*. (2007) Conserved factors regulate signalling in Arabidopsis thaliana shoot and root stem cell organizers. Nature, 446, 811–814. 17429400 10.1038/nature05703

[pbi12988-bib-0033] Sharma, S.K. , Millam, S. , Hedley, P.E. , McNicol, J. and Bryan, G.J. (2008) Molecular regulation of somatic embryogenesis in potato: an auxin led perspective. Plant Mol. Biol. 68, 185–201. 18553172 10.1007/s11103-008-9360-2

[pbi12988-bib-0034] Slotkin, R.K. , Vaughn, M. , Borges, F. , Tanurdzic, M. , Becker, J.D. , Feijo, J.A. and Martienssen, R.A. (2009) Epigenetic reprogramming and small RNA silencing of transposable elements in pollen. Cell, 136, 461–472. 19203581 10.1016/j.cell.2008.12.038 PMC2661848

[pbi12988-bib-0035] Stelpflug, S.C. , Eichten, S.R. , Hermanson, P.J. , Springer, N.M. and Kaeppler, S.M. (2014) Consistent and heritable alterations of DNA methylation are induced by tissue culture in maize. Genetics, 198, 209–218. 25023398 10.1534/genetics.114.165480 PMC4174933

[pbi12988-bib-0036] Stroud, H. , Ding, B. , Simon, S.A. , Feng, S. , Bellizzi, M. , Pellegrini, M. , Wang, G.L. *et al*. (2013) Plants regenerated from tissue culture contain stable epigenome changes in rice. Elife, 2, e00354. 23539454 10.7554/eLife.00354 PMC3601819

[pbi12988-bib-0037] Stroud, H. , Do, T. , Du, J. , Zhong, X. , Feng, S. , Johnson, L. , Patel, D.J. *et al*. (2014) Non‐CG methylation patterns shape the epigenetic landscape in Arabidopsis. Nat. Struct. Mol. Biol. 21, 64–72. 24336224 10.1038/nsmb.2735 PMC4103798

[pbi12988-bib-0038] Sun, Q. and Zhou, D.X. (2008) Rice jmjC domain‐containing gene JMJ706 encodes H3K9 demethylase required for floral organ development. Proc. Natl. Acad. Sci. USA, 105, 13679–13684. 18765808 10.1073/pnas.0805901105 PMC2533249

[pbi12988-bib-0039] Tanurdzic, M. , Vaughn, M.W. , Jiang, H. , Lee, T.J. , Slotkin, R.K. , Sosinski, B. , Thompson, W.F. *et al*. (2008) Epigenomic consequences of immortalized plant cell suspension culture. PLoS Biol. 6, 2880–2895. 19071958 10.1371/journal.pbio.0060302 PMC2596858

[pbi12988-bib-0040] Thibaud‐Nissen, F. , Shealy, R.T. , Khanna, A. and Vodkin, L.O. (2003) Clustering of microarray data reveals transcript patterns associated with somatic embryogenesis in soybean. Plant Physiol. 132, 118–136. 12746518 10.1104/pp.103.019968 PMC166958

[pbi12988-bib-0041] Tian, G. , Cheng, L. , Qi, X. , Ge, Z. , Niu, C. , Zhang, X. and Jin, S. (2015) Transgenic cotton plants expressing double‐stranded RNAs target HMG‐CoA Reductase (HMGR) gene inhibits the growth, development and survival of cotton bollworms. Int. J. Biol. Sci. 11, 1296–1305. 26435695 10.7150/ijbs.12463 PMC4582153

[pbi12988-bib-0042] Trapnell, C. , Williams, B.A. , Pertea, G. , Mortazavi, A. , Kwan, G. , van Baren, M.J. , Salzberg, S.L. *et al*. (2010) Transcript assembly and quantification by RNA‐Seq reveals unannotated transcripts and isoform switching during cell differentiation. Nat. Biotechnol. 28, 511–515. 20436464 10.1038/nbt.1621 PMC3146043

[pbi12988-bib-0043] Trapnell, C. , Roberts, A. , Goff, L. , Pertea, G. , Kim, D. , Kelley, D.R. , Pimentel, H. *et al*. (2012) Differential gene and transcript expression analysis of RNA‐seq experiments with TopHat and Cufflinks. Nat. Protoc. 7, 562–578. 22383036 10.1038/nprot.2012.016 PMC3334321

[pbi12988-bib-0044] Tu, L. , Zhang, X. , Liu, D. , Jin, S. , Cao, J. , Zhu, L. , Deng, F. *et al*. (2007) Suitable internal control genes for qRT‐PCR normalization in cotton fiber development and somatic embryogenesis. Chinese. Sci. Bull. 52, 3110–3117.

[pbi12988-bib-0045] Vining, K. , Pomraning, K.R. , Wilhelm, L.J. , Ma, C. , Pellegrini, M. , Di, Y. , Mockler, T.C. *et al*. (2013) Methylome reorganization during in vitro dedifferentiation and regeneration of Populus trichocarpa. BMC Plant Biol. 13, 92. 23799904 10.1186/1471-2229-13-92 PMC3728041

[pbi12988-bib-0046] Wang, S. , Chen, J. , Zhang, W. , Hu, Y. , Chang, L. , Fang, L. , Wang, Q. *et al*. (2015) Sequence‐based ultra‐dense genetic and physical maps reveal structural variations of allopolyploid cotton genomes. Genome Biol. 16, 108. 26003111 10.1186/s13059-015-0678-1 PMC4469577

[pbi12988-bib-0047] Wang, M. , Wang, P. , Tu, L. , Zhu, S. , Zhang, L. , Li, Z. , Zhang, Q. *et al*. (2016a) Multi‐omics maps of cotton fibre reveal epigenetic basis for staged single‐cell differentiation. Nucleic Acids Res. 44, 4067–4079. 27067544 10.1093/nar/gkw238 PMC4872108

[pbi12988-bib-0048] Wang, Q. , Zhu, Y. , Sun, L. , Li, L. , Jin, S. and Zhang, X. (2016b) Transgenic Bt cotton driven by the green tissue‐specific promoter shows strong toxicity to lepidopteran pests and lower Bt toxin accumulation in seeds. Sci. China Life Sci. 59, 172–182. 26728504 10.1007/s11427-015-4920-6

[pbi12988-bib-0049] Wang, P. , Zhang, J. , Sun, L. , Ma, Y. , Xu, J. , Liang, S. , Deng, J. *et al*. (2017) High efficient multisites genome editing in allotetraploid cotton (Gossypium hirsutum) using CRISPR/Cas9 system. Plant Biotechnol. J. 16, 137–150. 28499063 10.1111/pbi.12755 PMC5785356

[pbi12988-bib-0050] Wilkins, T.A. , Rajasekaran, K. and Anderson, D.M. (2000) Cotton biotechnology. Crit. Rev. Plant Sci. 19, 511–550.

[pbi12988-bib-0051] Wu, J. , Zhang, X. , Nie, Y. , Jin, S. and Liang, S. (2004) Factors affecting somatic embryogenesis and plant regeneration from a range of recalcitrant genotypes of Chinese cottons (Gossypium hirsutum L.). In Vitro Cell & Dev. Biol‐Plant. 40, 371–375.

[pbi12988-bib-0052] Yang, X. and Zhang, X. (2010) Regulation of somatic embryogenesis in higher plants. Crit. Rev. Plant Sci. 29, 36–57.

[pbi12988-bib-0053] Yang, X. , Zhang, X. , Yuan, D. , Jin, F. , Zhang, Y. and Xu, J. (2012) Transcript profiling reveals complex auxin signalling pathway and transcription regulation involved in dedifferentiation and redifferentiation during somatic embryogenesis in cotton. BMC Plant Biol. 12, 110. 22817809 10.1186/1471-2229-12-110 PMC3483692

[pbi12988-bib-0054] Yang, X. , Wang, L. , Yuan, D. , Lindsey, K. and Zhang, X. (2013) Small RNA and degradome sequencing reveal complex miRNA regulation during cotton somatic embryogenesis. J. Exp. Bot. 64, 1521–1536. 23382553 10.1093/jxb/ert013 PMC3617824

[pbi12988-bib-0055] Zakrzewski, F. , Schmidt, M. , Van Lijsebettens, M. and Schmidt, T. (2017) DNA methylation of retrotransposons, DNA transposons and genes in sugar beet (Beta vulgaris L.). Plant. J. 90, 1156–1175. 28257158 10.1111/tpj.13526

[pbi12988-bib-0056] Zemach, A. , Kim, M.Y. , Hsieh, P.H. , Coleman‐Derr, D. , Eshed‐Williams, L. , Thao, K. , Harmer, S.L. *et al*. (2013) The arabidopsis nucleosome remodeler DDM1 Allows DNA methyltransferases to access H1‐containing heterochromatin. Cell, 153, 193–205. 23540698 10.1016/j.cell.2013.02.033 PMC4035305

[pbi12988-bib-0057] Zhang, Y. , Liu, T. , Meyer, C.A. , Eeckhoute, J. , Johnson, D.S. , Bernstein, B.E. , Nussbaum, C. *et al*. (2008) Model‐based analysis of ChIP‐Seq (MACS). Genome Biol. 9, R137. 18798982 10.1186/gb-2008-9-9-r137 PMC2592715

[pbi12988-bib-0058] Zhang, T. , Hu, Y. , Jiang, W. , Fang, L. , Guan, X. , Chen, J. , Zhang, J. *et al*. (2015) Sequencing of allotetraploid cotton (Gossypium hirsutum L. acc. TM‐1) provides a resource for fiber improvement. Nat. Biotechnol. 33, 531–537. 25893781 10.1038/nbt.3207

[pbi12988-bib-0059] Zhong, S. , Fei, Z. , Chen, Y.R. , Zheng, Y. , Huang, M. , Vrebalov, J. , McQuinn, R. *et al*. (2013) Single‐base resolution methylomes of tomato fruit development reveal epigenome modifications associated with ripening. Nat. Biotechnol. 31, 154–159. 23354102 10.1038/nbt.2462

