# Supplementary material for: Multi‐omics analyses reveal epigenomics basis for cotton somatic embryogenesis through successive regeneration acclimation process
Source: Plant Biotechnol J. 2018 Sep 30;17(2):435–50. doi: 10.1111/pbi.12988 (PMC6335067; doi:10.1111/pbi.12988)
Supplement: Supplementary file 1 — Figure S1 CG, CHG and CHH methylation patterns of TEGs and PCGs. Figure S2 The numbers of hypomethylated (Hypo) and hypermethylated (Hyper) context‐specific differentially methylated regions (DMRs) in regenerated plants. Figure S3 A circos plot of the TE density, gene density, CG‐differentially methylated region (DMR), CHG‐DMR, and CHH‐DMR in comparisons of WT_vs_R0, R0_vs_R2 and R2_vs_R4. The hyper‐DMRs and hypo‐DMRs were normalized by the density. The red and green colors represent hyper‐DMRs and hypoDMRs, respectively. Figure S4 The number of overlapped CG‐, CHG‐ and CHH‐DMRs in the three groups. Figure S5 An example of hypermethylation in the comparison of R0_vs_R2. Every orange triangle represents one DMR. Figure S6 CHH methylation level in different TE families. Figure S7 The distribution of TEs length. The upper panel are short TE (<0.5‐kb), middle TE (0.5‐4‐kb), and long TE (>4‐kb) according to TE length. The long TE divide into long Gypsy, long Copia and the others (under panel). Figure S8 Average distribution of CHH methylation in short TEs and long TEs with their flanking 2‐kb regions. Figure S9 The RdDM effects in gene body region during the somatic embryogenesis process. Figure S10 The circos plot shows the TE density, gene density, CG‐DMR, CHGDMR and CHH‐DMR in NEC_vs_EC, EC_vs_SE and SE_vs_R0 groups. Figure S11 The numbers of overlapped CG‐, CHG‐, CHH‐DMRs during tissue culture development stages and from WT to regeneration plants. Figure S12 Gene expression have weak negatively correlation with DNA methyl of promoter 2‐kb. [file PBI-17-435-s004.pdf]

Supporting Figures

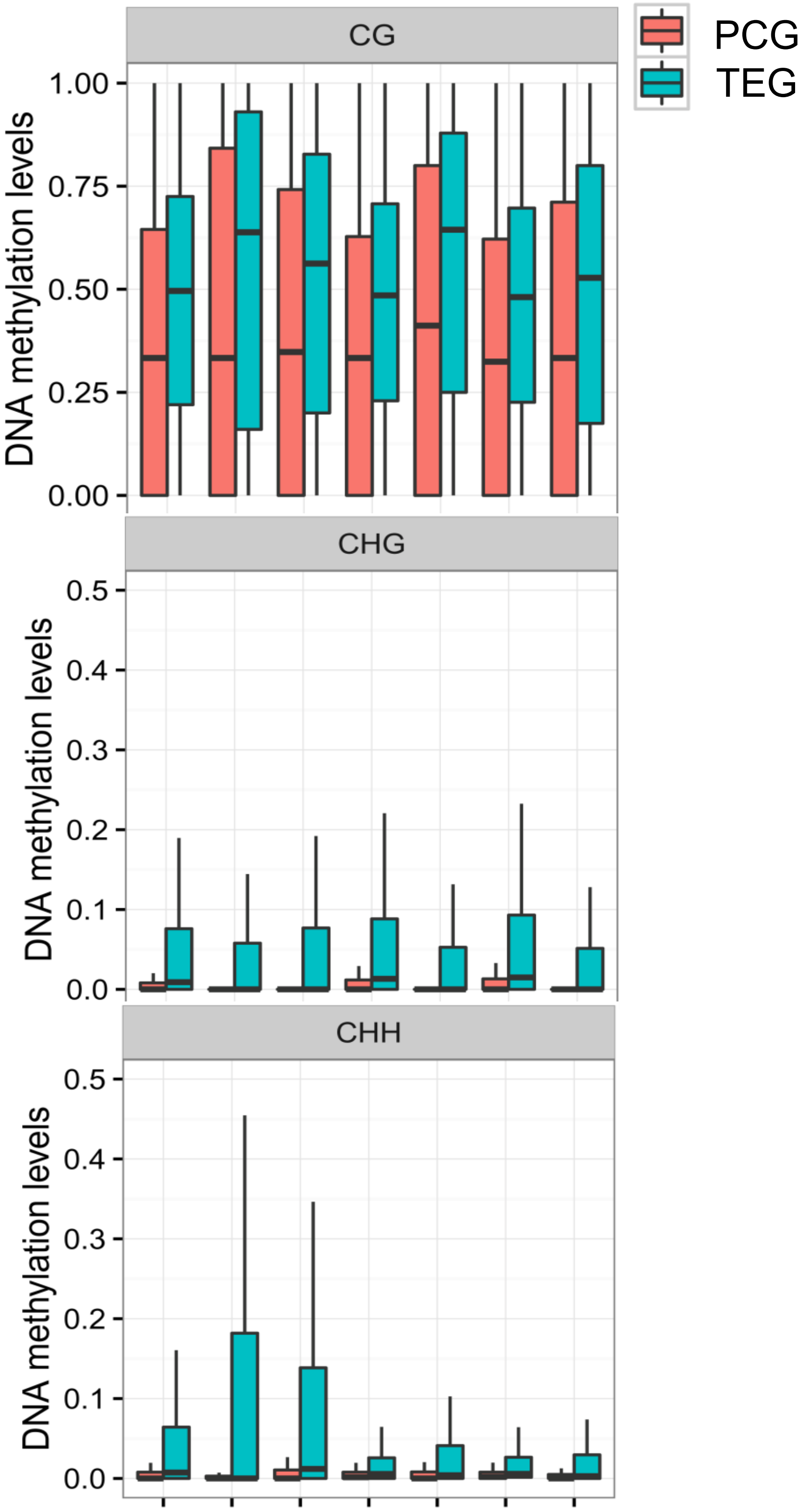

Figure S1 CG, CHG and CHH methylation patterns of TEGs and PCGs.

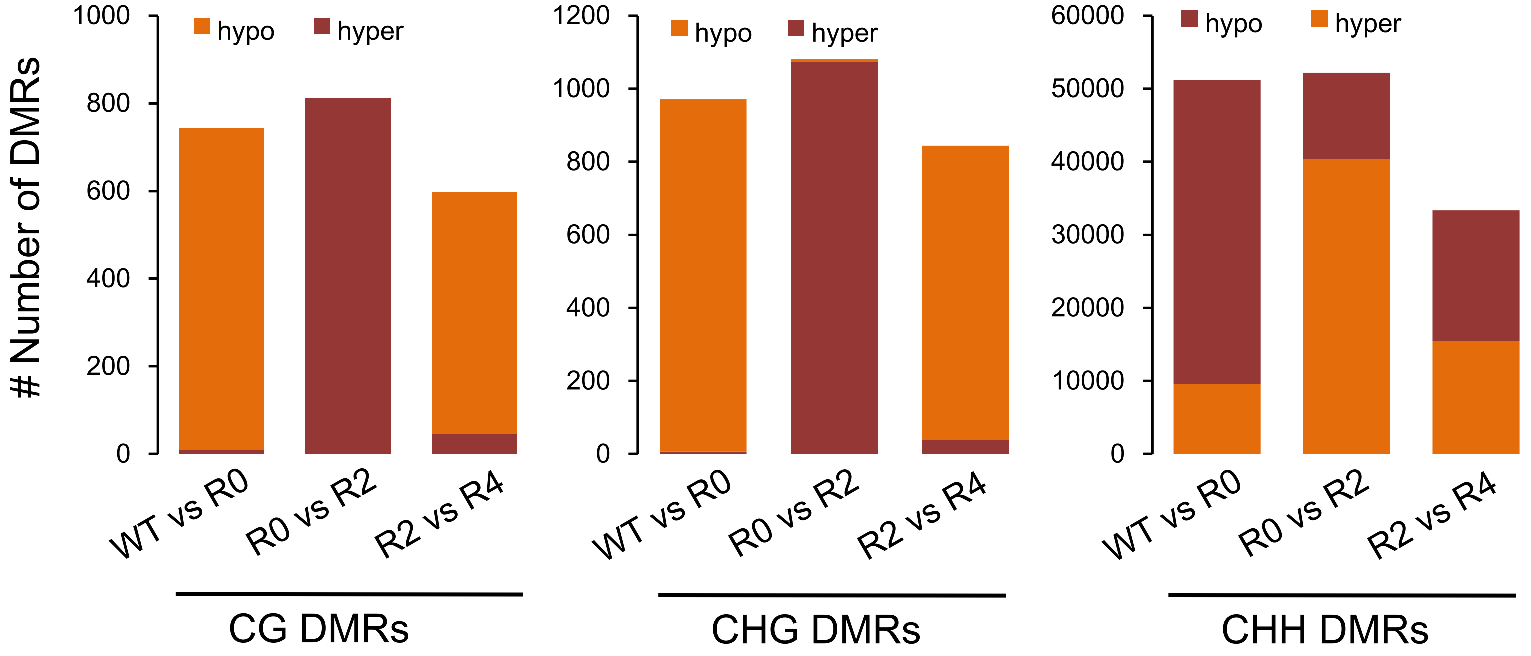

Figure S2 The numbers of hypomethylated (Hypo) and hypermethylated (Hyper) context-specific differentially methylated regions (DMRs) in regenated plants.

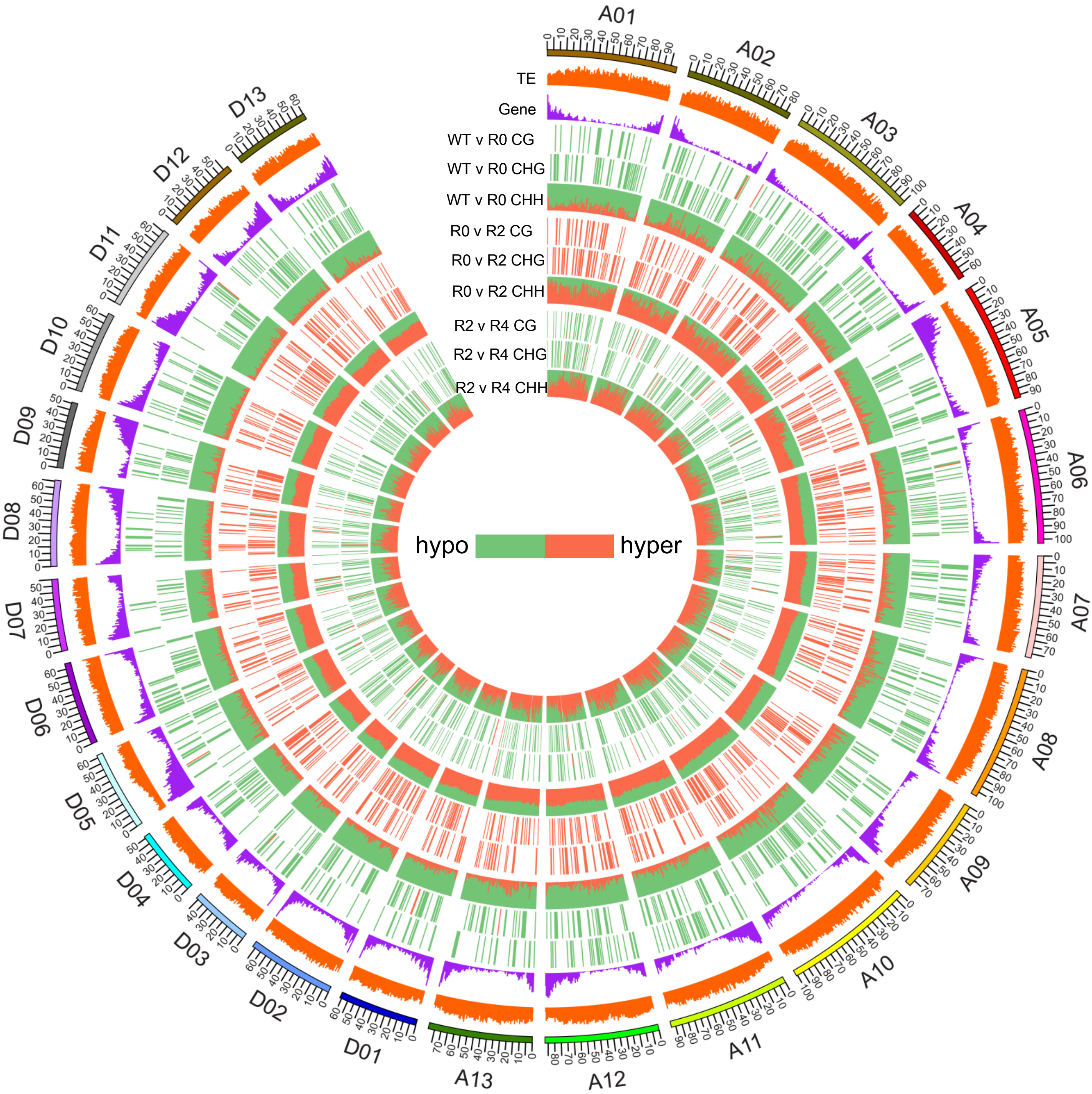

Figure S3 A circos plot of the TE density, gene density, CG-differentially methylated region (DMR), CHG-DMR, and CHH-DMR in comparisons of WT_vs_R0, R0_vs_R2 and R2_vs_R4. The hyper-DMRs and hypo-DMRs were normalized by the density. The red and green colors represent hyper-DMRs and hypo-DMRs, respectively.

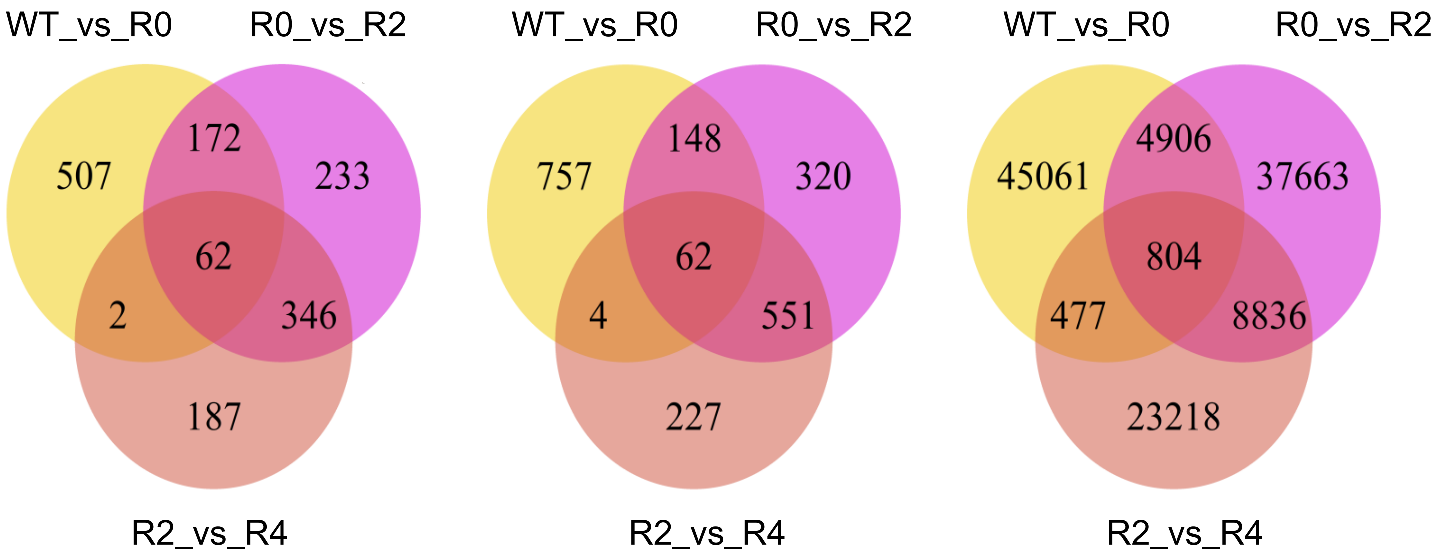

Figure S4 The number of overlapped CG-, CHG- and CHH-DMRs in the three groups.

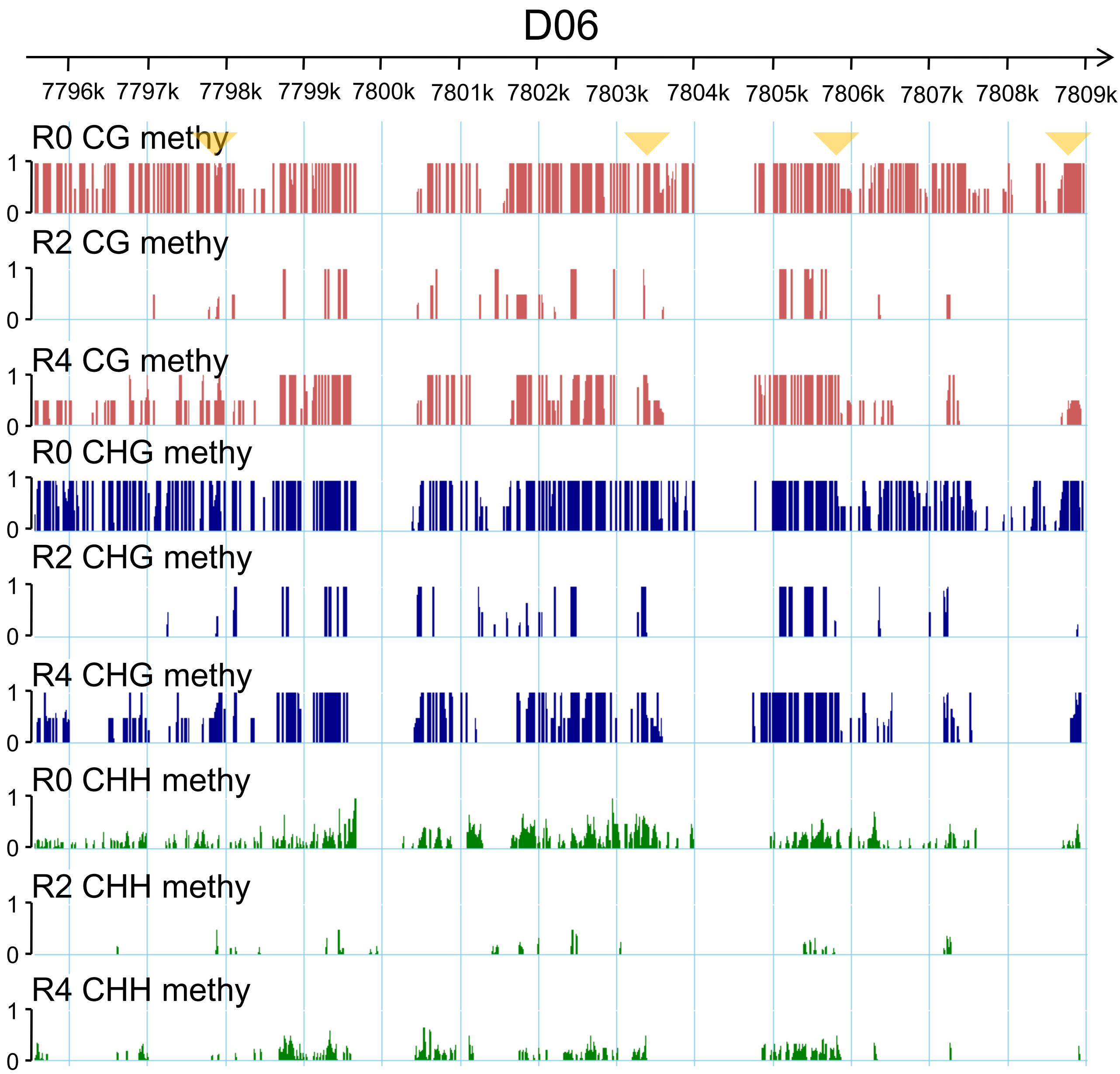

Figure S5 An example of hypermethylation in the comparison of R0_vs_R2. Every orange triangle represents one DMR.

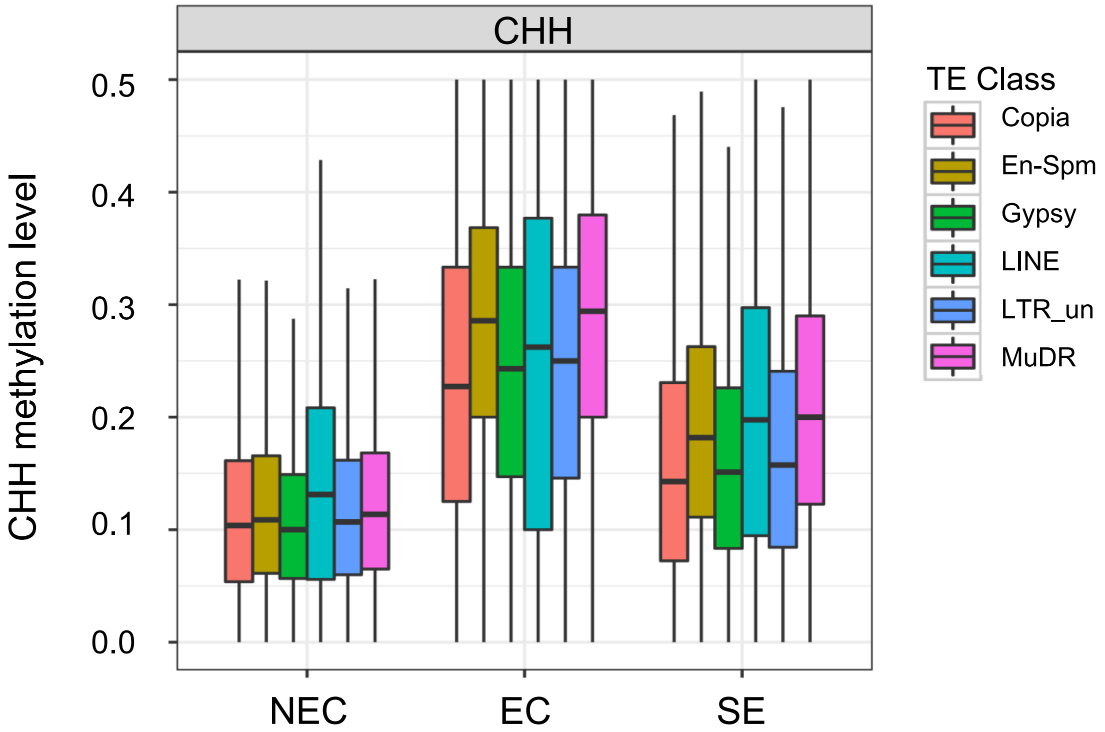

Figure S6 CHH methylation level in different TE families during the somatic embryogenesis process

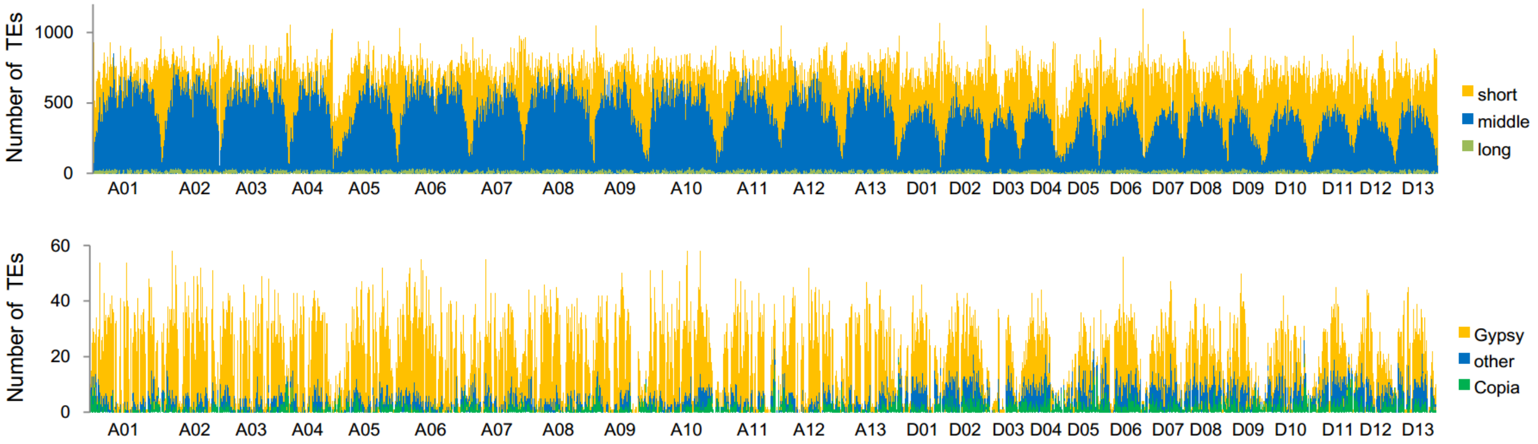

Figure S7 The distribution of TEs length. The upper panel are short TE (<0.5kb), middle TE (0.5-4kb), and long TE (>4kb) accroding to TE length. The long TE divide into long Gypsy, long Copia, and the others (under panel).

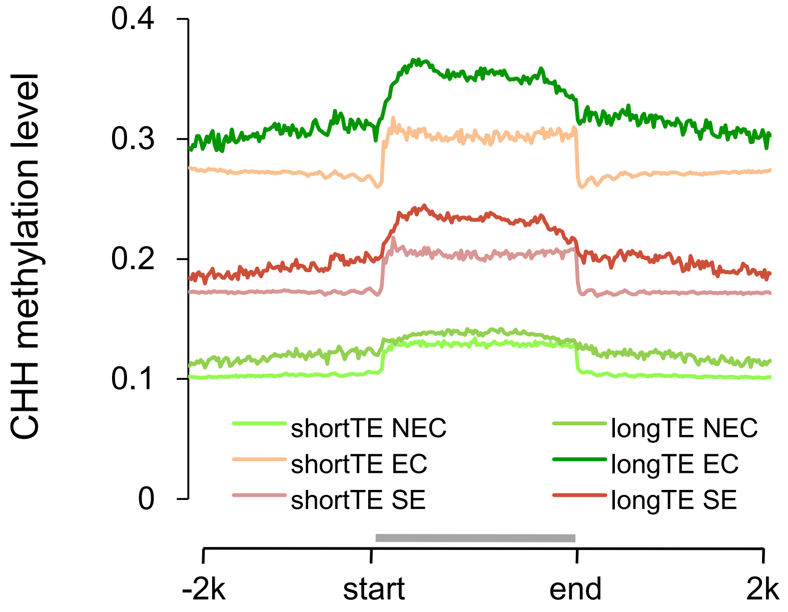

Figure S8 Average distribution of CHH methylation in short TEs and long TEs with their flanking 2-kb regions.

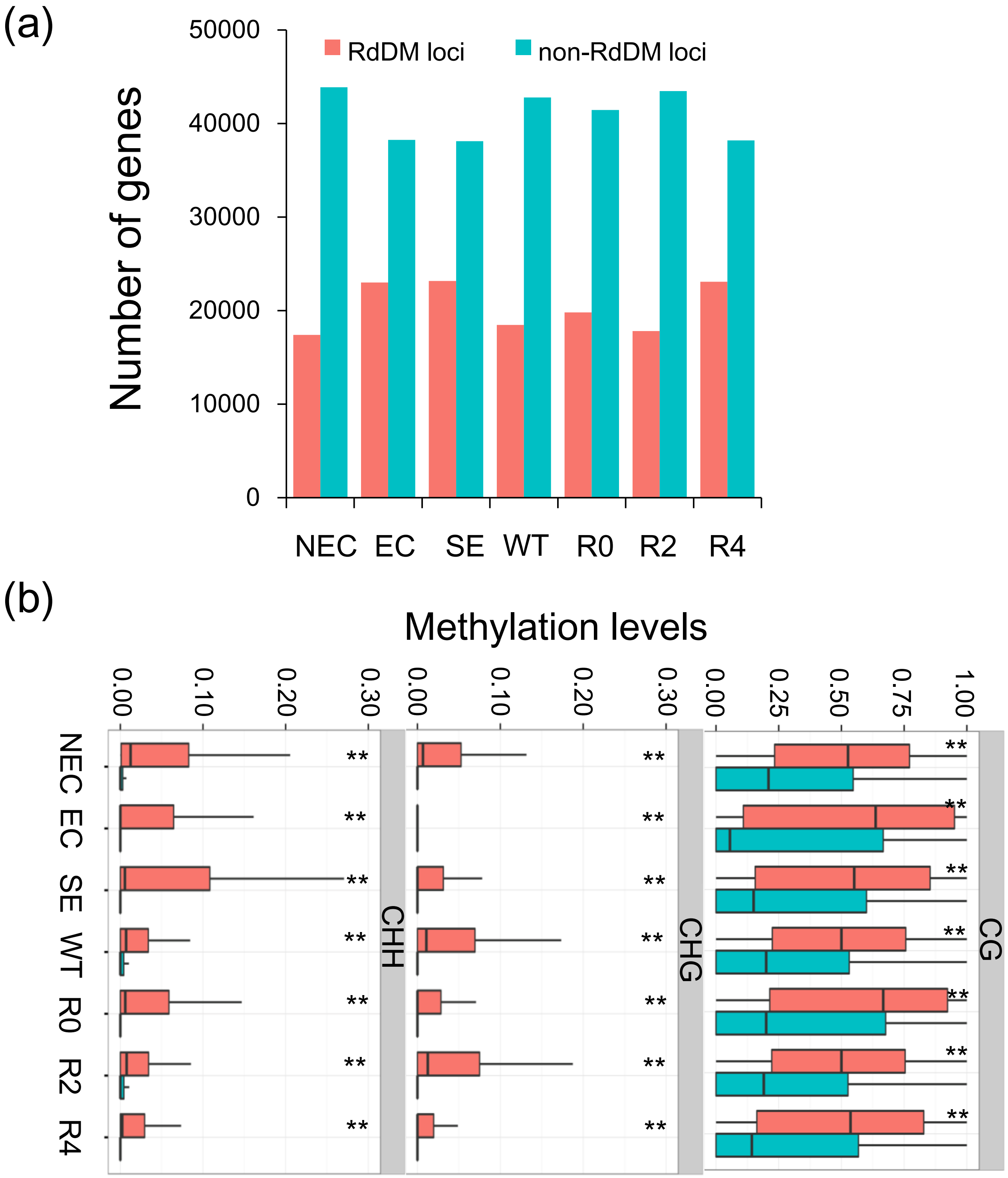

Figure S9 The RdDM effects in gene body region during the somatic embryogenesis process. (a)The number of RdDM loci overlapped (red) and non-overlapped (green) genes. (b) The DNA methylation patterns of RdDM loci marked genes and non-RdDM loci marked genes in the tissue culture process and regenerated plants.

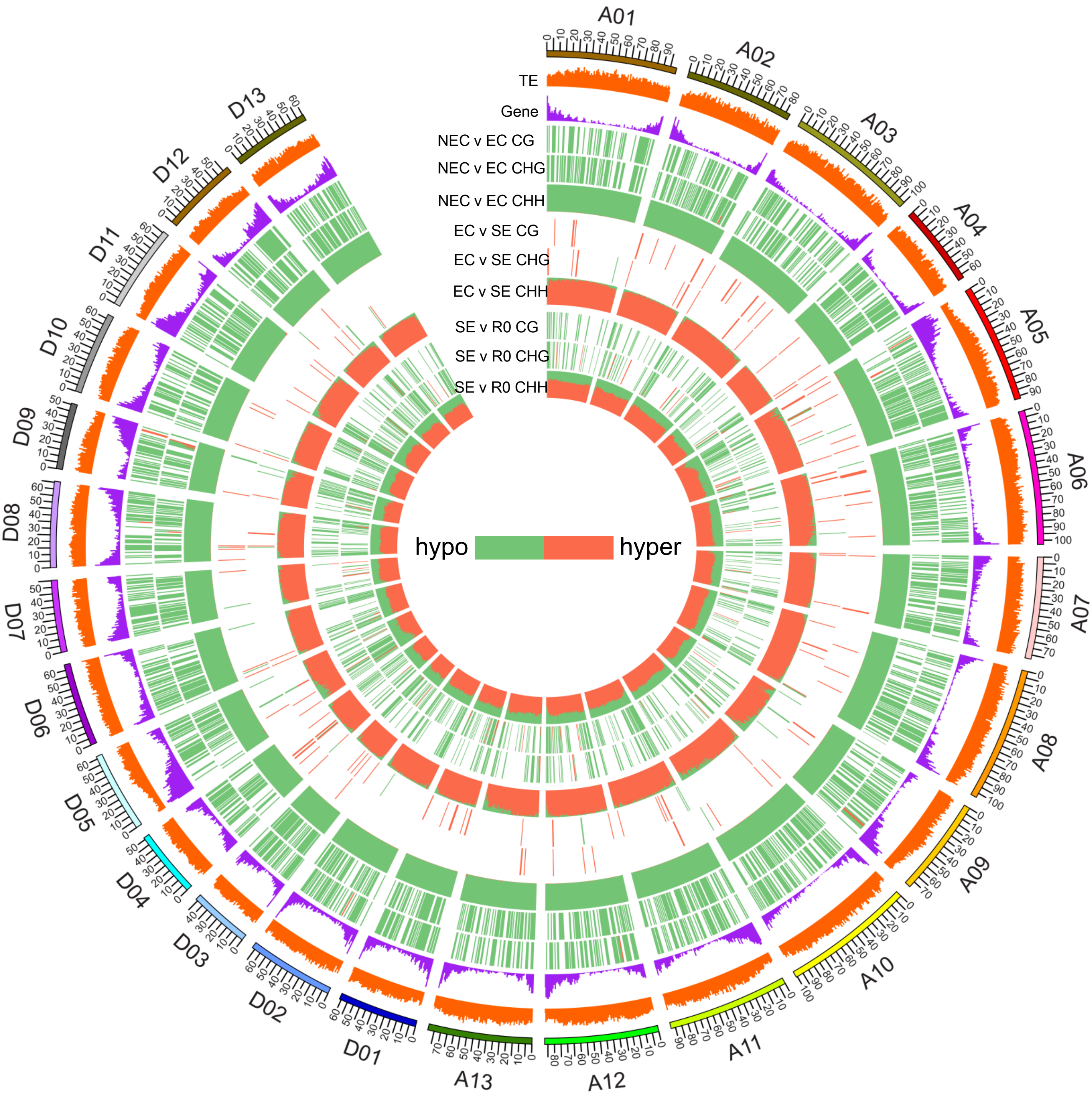

Figure S10 The circos plot shows the TE density, gene density, CG-DMR, CHG-DMR and CHH-DMR in NEC_vs_EC, EC_vs_SE and SE_vs_R0 groups.

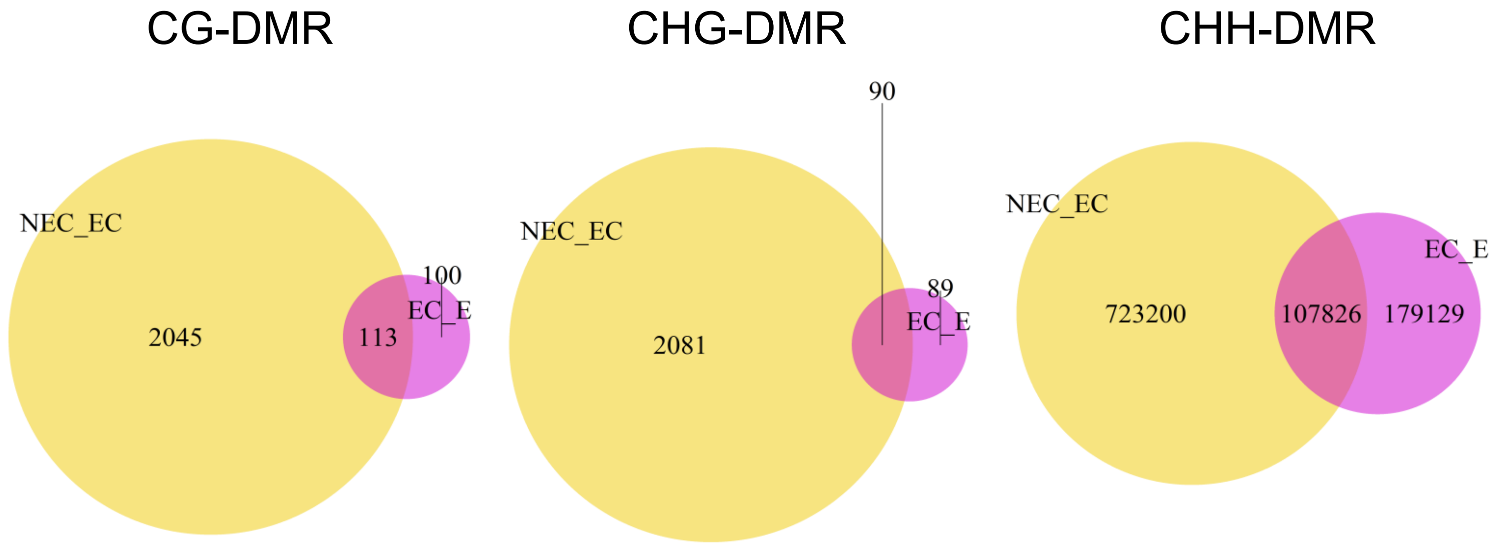

Figure S11 The numbers of overlapped CG-, CHG-, CHH-DMRs during tissue culture development stages and from WT to regeneration plants.

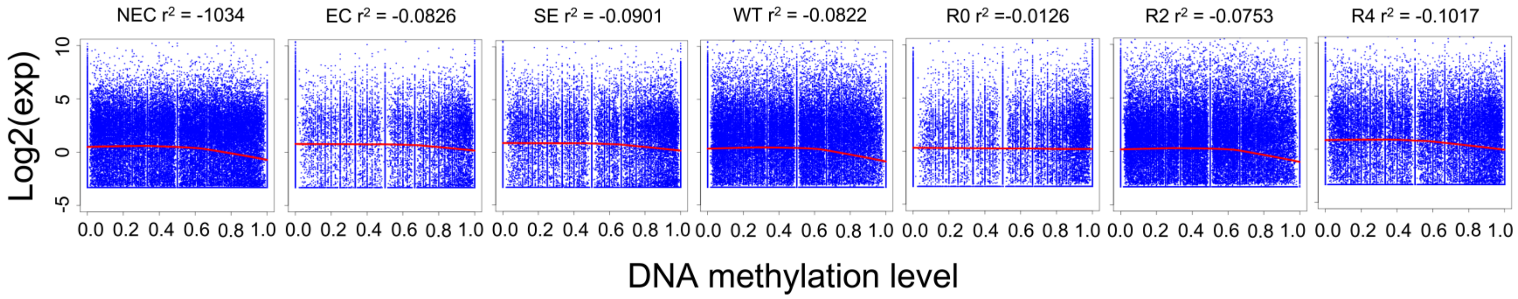

Figure S12 Gene expression have weak negatively correlation with DNA methy lation of gene promoter 2KB. The 'Log2(exp) ' represent the normalized the gene expression.
