# Supplementary material for: Multi‐omics analyses reveal epigenomics basis for cotton somatic embryogenesis through successive regeneration acclimation process
Source: Plant Biotechnol J. 2018 Sep 30;17(2):435–50. doi: 10.1111/pbi.12988 (PMC6335067; doi:10.1111/pbi.12988)
Supplement: Supplementary file 2 — Table S1 Summary of BS‐Seq data in G. hirsutum. Table S2 Identification of methylated cytosines in each of the seven samples. Table S3 Summary of small RNA‐Seq data. Table S4 Summary of H3K9me2 data. Table S5 Summary of the full names of DNA methylation pathway related genes. Table S6 Summary of RNA‐Seq data. Table S7 Summary of RNA‐Seq data from mock and zebularine treatment of callus. Table S8 The expression patterns of RdDM related genes and DNA methyltransferases under zebularine treatment. [file PBI-17-435-s005.pdf]

Supporting Tables

Table S1 Summary of BS-Seq data in *G.hirsutum*.

Samples	Raw reads	Clean reads	Depth	Coverage (>3)	Unique mapping	SRA accession
NEC	169,963,719	143,061,716	13.6	322,454,486	70.50%	SRR5527420
EC	170,884,736	139,309,348	13.67	210,720,869	75.70%	SRR5527422
SE	170,885,236	138,210,842	13.67	265,407,542	72.70%	SRR5527423
WT	160,863,889	115,480,444	12.87	236,595,542	59.10%	SRR5527460
R0	182,884,232	138,456,249	14.63	291,773,956	73.10%	SRR5527461
R2	132,406,207	111,063,155	10.59	241,982,385	67.10%	SRR5527462
R4	180,884,336	149,275,437	14.47	265,927,442	72.40%	SRR5527463

Table S2 Identifcation of methylated cytosines in each of the seven samples.

Type	NEC	EC	SE	WT	R0	R2	R4
mCG	36,198,946	28,476,574	32,530,275	24,060,024	35,968,628	24,047,701	32,388,673
mCHG	33,461,047	30,169,229	32,567,664	20,609,240	35,519,046	20,681,707	31,435,968
mCHH	26,075,746	152,075,066	38,802,630	9,518,666	19,900,255	10,071,516	12,883,370

Table S3 Summary of small RNA-Seq data.

Sample	Raw reads	uniq reads	24-nt mapping	SRA accession
NEC_Rep1	10,988,643	942,251	90.83%	SRR5527300
NEC_Rep2	10,789,959	2,483,594	73.31%	SRR5527301
EC_Rep1	11,246,664	5,115,114	75.30%	SRR5527302
EC_Rep2	11,170,465	4,702,214	74.32%	SRR5527303
SE_Rep1	11,033,946	5,124,031	75.81%	SRR5527304
SE_Rep2	11,303,312	5,098,147	76.36%	SRR5527413
WT_Rep1	11,380,515	4,215,399	69.57%	SRR5527452
WT_Rep2	10,041,102	1,949,794	89.15%	SRR5527453
R0_Rep1	10,014,283	2,801,352	91.31%	SRR5527454
R0_Rep2	11,481,165	5,501,111	75.54%	SRR5527455
R2_Rep1	10,036,477	2,217,621	89.49%	SRR5527456
R2_Rep2	11,101,114	5,166,868	73.49%	SRR5527457
R4_Rep1	11,344,495	3,969,251	73.65%	SRR5527458
R4_Rep2	11,319,537	4,209,981	75.16%	SRR5527459

Table S4 Summary of H3K9me2 data.

Samples	Raw reads	Clean reads	H3K9me2 peaks	SRA accession
NEC_H3K9me2	34,456,614	26,636,054	23,225	SRR5527424
EC_H3K9me2	28,920,348	26,528,132	22,892	SRR5527424
SE_H3K9me2	29,440,811	21,579,511	39,838	SRR5527424

Table S5 Summary of the full names of DNA methylation pathway related genes.

Gossspium hirsutum			
Gene symbol	Gene Name	A-genome	D-genome
MET1	methyltransferases 1	Gh_A05G3224,	Gh_D04G0381
CMT3	chromomethylase 3	Gh_A07G0385,Gh_A13G2087	Gh_D07G0449
CMT2	chromomethylase 2	Gh_A08G1371,Gh_A08G1372	Gh_D08G1665,Gh_D08G1666
AGO4	Argonaute family protein 4	Gh_A08G1752	Gh_D08G2707
DCL3	dicer-like 3		Gh_D06G0845,Gh_D13G2027
RDR2	RNA-dependent RNA polymerase 2	Gh_A12G2496	Gh_D12G2624
DDM1	decreased DNA methylation 1	Gh_A12G0603	Gh_D12G2782
NRP	nuclear RNA polymerase A/C		Gh_D03G0635,Gh_D11G0040
DRM	DNA glycosylase DEMETER		
ROS1	repressor of silencing 1	Gh_A04G0555	Gh_D04G1010

Table S6 Summary of RNA-Seq data.

Samples	Raw reads	Clean reads	mapping	SRA accession
NEC_Rep1	27,374,440	22,333,987	92.60%	SRR5527245
NEC_Rep2	25,409,326	22,236,782	92.50%	SRR5527246
EC_Rep1	42,701,596	37,990,391	96.50%	SRR5527247
EC_Rep2	31,225,317	27,574,399	92.50%	SRR5527248
SE_Rep1	59,636,331	47,429,531	94.30%	SRR5527249
SE_Rep2	26,570,105	23,483,786	92.00%	SRR5527250
WT	27,379,640	22,696,970	90.50%	SRR5527434
R0	28,276,570	23,238,678	90.30%	SRR5527435
R2	30,308,644	24,877,011	92.60%	SRR5527436
R4	51,516,953	20,822,106	85.00%	SRR5527437

Table S7 Summary of RNA-Seq data from mock and zebularine treatment of callus.

Samples	Raw reads	Clean reads	Unpaired reads	MappingGh	SRA accession
mock_Rep1	17,509,741	15,533,491	1,568,118	91.80%	SRR5383896
mock_Rep2	12,895,037	11,229,712	1,357,530	91.60%	SRR5383897
100um_Rep1	15,226,047	13,392,882	1,465,685	91.70%	SRR5383898
100um_Rep2	14,552,249	12,798,035	1,344,958	91.80%	SRR5383899

Table S8 The expression patterns of RdDM related genes and DNA methyltransferases under zebularine treatment.

symbol	gene_id	mock_Rep1	mock_Rep2	100um_Rep1	100um_Rep2
CMT2	Gh_D08G1665	3.22386	4.07339	3.42361	3.33926
	Gh_D08G1666	8.86877	10.1301	10.2171	13.2116
	Gh_A08G1371	2.57523	2.44502	2.40607	2.3281
	Gh_A08G1372	6.86816	6.52455	6.01481	5.3889
DCL3	Gh_D06G0845	3.63062	4.213	4.35725	4.22331
	Gh_D13G2027	0.114138	0.113714	0.32356	0.198201
DDM1	Gh_A12G0603	1.30536	1.37174	1.56663	2.02044
	Gh_D12G2782	0.625157	1.3824	1.96155	2.1153
RDR2	Gh_D12G2624	2.01071	2.25415	2.89741	2.38717
	Gh_A12G2496	1.84548	1.93774	1.72228	1.3617
NRPD	Gh_A11G0189	0.0395028	0.0275732	0.0230648	0.0479846
	Gh_D08G1916	1.39079	1.1665	1.28882	0.997748
	Gh_A08G1605	1.04626	1.14978	1.03578	1.00115
	Gh_D11G0200	1.90238	2.27749	1.57159	0.900469
DRM1	Gh_A09G0264	6.95835	7.44438	9.07887	7.71594
	Gh_D09G0266	7.17104	7.08213	8.95675	6.2013
	Gh_A05G3114	0.780303	0.492613	0.456679	0.425843
	Gh_D04G0527	4.65802	4.17598	2.74289	2.32056
AGO4	Gh_A08G1752	37.0492	36.1401	43.4166	36.6895
	Gh_D08G2707	23.0665	23.492	32.5425	28.8187
MET1	Gh_A05G3224	3.36949	3.77256	2.45366	2.03673
	Gh_D04G0381	11.5964	12.4232	4.00875	3.64981
CMT3	Gh_D07G0449	0.480342	0.485296	0.373776	0.355858
	Gh_A07G0385	0.876912	0.705517	0.432972	0.546233
	Gh_A13G2087	3.55864	3.44512	3.63835	3.27368
ROS1	Gh_D04G1010	9.78204	12.0622	9.46397	8.26991
	Gh_D09G1341	0.416767	0.629581	0.360521	0.332592
	Gh_A04G0555	9.46008	12.1205	8.63702	7.64892
	Gh_A09G2457	0.660272	0.680856	0.470501	0.282463
DME	Gh_D01G1149	1.11586	1.05176	1.37154	1.3126
	Gh_A01G1068	0.53836	0.579204	0.327262	0.474756
	Gh_D05G0847	0.180301	0.347534	0.30667	0.319693
	Gh_A05G0700	0.682124	0.910328	0.910421	0.741722

Data S1 The expression and mCHH methylation encoding RdDM pathway genes.

Data S2 The hyper-DMRs (promoter 2-kb) in ECs and corresponding gene expression from NEC to EC.

Data S3 The expression and mCHH methylation hormone-related genes somatic embryogenesis process.
